# Multi-Level Safety Studies of Anti Fel d 1 IgY Ingredient in Cat Food

**DOI:** 10.3389/fvets.2019.00477

**Published:** 2020-01-08

**Authors:** Ray A. Matulka, Larry Thompson, David Corley

**Affiliations:** ^1^Burdock Group Consultants, Orlando, FL, United States; ^2^Nestlé Purina PetCare Global Resources, Inc., St. Louis, MO, United States

**Keywords:** feline, IgY, Fel d 1, allergenic, genotoxicity

## Abstract

Chickens exposed to antigens produce IgY antibodies, similar in structure to mammalian IgG. Hens exposed with an allergen produced by cats (Fel d 1) results in production of anti-Fel d 1 specific IgY (AFD1), which is naturally concentrated in egg yolk. A chicken egg product ingredient containing AFD1 was evaluated for safety in a 26-week randomized, controlled, blinded tolerance study in cats and *in vitro* for mutagenic and genotoxic effects. The *in vivo* study was conducted with groups fed kibble containing 0, 7, 39, or 66 ppm AFD1. Parameters examined included: clinical observations, body weights, food consumption, serum chemistry, hematology, blood coagulation, urinalyses, and mortality and morbidity checks. AFD1 was evaluated for potential mutagenic effects utilizing the bacterial reverse mutation assay at concentrations of up to 2.78 ppm and for potential structural chromosomal aberrations at up to 3 ppm using human peripheral blood lymphocytes (HPBL). After 6-months of feeding to cats, there were no significant differences between control and any test groups in any parameters analyzed. No significant increases in mutations or chromosomal aberrations were observed in tests with or without metabolic activation (S9). These studies show AFD1 was well-tolerated in cats at levels tested and does not induce mutagenic or chromosomal aberrations under study conditions.

## Introduction

Pet ownership has been steady or increasing in the United States and worldwide, with cats being the second-most frequent household pet. However, sensitization to cat allergens is estimated at ~12.1% of the population 6 years old or older (1). The major cat allergen is the secretoglobin Fel d 1, a protein of unknown function produced by the skin and by salivary and lacrimal glands of cats (2–5). Fel d 1 produced in the saliva is distributed onto the cat's hair through grooming, and dispersed into the environment on shed hair and dander (dried skin cells).

The pet food industry is focused on the health and well-being of pets and their relationship with their owners, developing innovative ways for people and their pets to live better lives. For example, the chicken (*Gallus gallus domesticus*) has been raised for meat and eggs for at least 3,000 years (6), but only relatively recently have components of the egg been identified and tested to benefit cats and other animals.

The egg is a unique complex feed ingredient that contains the nutrients required to support the chicken's early life including immunologically active immunoglobulin (Ig)Y (IgY) antibody proteins (7, 8). An ingredient has been developed for use in cat food that contains IgY antibodies directed toward the Fel d 1 antigen (AFD1). The ingredient is coated onto dry cat food and as the food is chewed and eaten, the AFD1 binds multiple epitopes of Fel d 1 in the cat's saliva. The bound and neutralized Fel d 1 is then spread to the cat's hair through grooming and released into the environment. Whereas unbound, *active* Fel d 1 is a potent allergen, Fel d 1 bound by AFD1 is unable to bind to IgE and is not recognized as an allergen by the sensitized human. This novel approach to reducing allergenic Fel d 1 exposure by use of AFD1 was recently evaluated in a 10-week feeding period in which cats consumed a food containing the anti-Fel d 1 IgY-containing AFD1 ingredient (9). Consumption of AFD1 significantly reduced the active Fel d 1 on the cat's hair, with the cats producing the greatest amount of Fel d 1 demonstrating the greatest decrease in Fel d 1 on the hair.

Chickens naturally produce IgY in response to exposure to antigens in their environment, and all egg products contain IgY (7). The AFD1 ingredient is in a unique category: while IgY-containing egg products have been frequently included in cats' diets for many years, a commercially-produced egg yolk product containing antibodies directed toward the Fel d 1 protein has not been previously marketed and the daily effect of binding the secreted Fel d 1 protein is currently unknown. New ingredients or ingredients with novel properties must undergo a rigorous safety assessment under the intended conditions of use prior to commercial release pet food (10). To this end, studies were conducted to ensure the safety of AFD1 for use in cat food. Reported in this paper are the results of a 26-week multi-level tolerance study in cats and evaluation of the potential for genotoxicity by the ingredient using standard *in vitro* methods.

## Materials and Methods

### Test Feeding Ingredient

An egg product ingredient containing IgY immunoglobulins specific for Fel d 1 antigen was provided by Nestlé Purina PetCare Global Resources, Inc. The egg product ingredient is an off-white, granular processed egg yolk powder with a maximum 5% moisture, greater than 28% protein and a maximum 7% ash, providing at least 1,000 parts per million (ppm) Anti-fel d1 IgY.

### Chemicals and Materials

The bacterial reverse mutation assay utilized 2-aminoanthracene (2-AA), 2-nitrofluorene (2-NF), sodium azide (SA), 9-aminoacridine (9-AAD), methyl methanesulfonate (MMS), dimethylsulfoxide (DMSO), and water obtained from Sigma-Aldrich (Saint Louis, MO). The Aroclor 1254-induced rat liver S9 metabolic activation mixture was purchased from MolTox® (Boone, NC). Media components used for this assay included D-biotin, L-histidine (0.5 mM), BBL select agar, and L-tryptophan, Oxoid No. 2 nutrient agar and broth, and custom top agar (all from MolTox®). The control vehicle was sterile filtered bioreagent water (Sigma-Aldrich, St. Louis, MO).

The *in vitro* mammalian human peripheral blood lymphocyte (HPBL) chromosomal aberration assay used water (Ricerca BioSciences; Concord, OH), mitomycin C (MMC) (Sigma-Aldrich), cyclophosphamide (CP) (Sigma-Aldrich), and sterile distilled water for dilution (Thermo Fisher Scientific; Waltham, MA).

### Feline Diet

Prior to randomization for study use, the cats were transitioned from a standard laboratory diet^1^ to a commercial chicken and rice adult dry cat food diet according to a veterinary directive. Four test diets were produced by Nestlé Purina PetCare Global Resources, Inc. The AFD1 ingredient was blended with a flavoring system and then applied to the control diet that provided AFD1 at levels of 0 ppm (control), 7 ppm, 39 ppm, 66 ppm, respectively. Starting on Day 1, all cats were fed their assigned diet in amounts needed to meet their daily energy requirement, determined by using their most recent body weight.

### Cats and Organisms

*Salmonella typhimurium* (derived from Dr. Bruce Ames' cultures) and *Escherichia coli* (from the National Collection of Industrial and Marine Bacteria, Aberdeen, Scotland) tester strains were used in the bacterial reverse mutation assay performed at BioReliance (Rockville, MD). The chromosomal aberration assay utilized HPBL obtained from a healthy non-smoking human male (30 years of age).

The dietary study utilized a group of 42 healthy adult domestic cats consisting of 21 males and 21 females. The cats were 1–3 years of age at the start of the study, with body weights ranging from 2.4 to 6.1 kg. The males were neutered and the females were intact, nulliparous, and non-pregnant. The cats were assigned a unique identification number prior to randomization that was used to identify all records and specimens derived from each cat. Prior to the start of the study, the cats were vaccinated at least once for rabies and at least twice (three weeks apart or more) with vaccines for feline rhinotracheitis, calicivirus, panleukopenia, and *Chlamydia psittaci*. The cats had not received any medication except for vaccinations within 30 days of Study Day 1.

### Experimental Design

#### Bacterial Reverse Mutation Assays

The bacterial reverse mutation assay was used to evaluate the ability of the AFD1 to induce point mutations in five strains of *Salmonella typhimurium* and one *Escherichia coli* strain (WP2 *uvr*A), in both the presence and absence of an S9 exogenous metabolic activation system, according to standard protocols (OECD 471). The assay was performed under current Good Laboratory Practices (cGLP) in accordance with Chapter 21 of the Code of Federal Regulations (CFR) Section 58 (GLP for non-clinical laboratory studies) and followed the Organization for Economic Co-operation and Development (OECD) guidance 471 (July, 1997).

The bacterial assay was conducted in two phases: an initial cytotoxicity-mutation assay that indicated a concentration range for the confirmation assay, and a subsequent confirmatory mutagenicity assay that used S. *typhimurium* tester strains TA98, TA100, TA1535, and TA1537 (Discovery Partners International, San Diego, CA) and *E. coli* tester strain WP2 *uvr*A (National Collection of Industrial and Marine Bacteria, Aberdeen, Scotland) to ascertain AFD1 product-induced mutation potential. For the initial toxicity study, concentrations of AFD1 at 0.00083, 0.00278, 0.0083, 0.0278, 0.083, 0.278, 0.83, 2.78 ppm were prepared in water, the vehicle control. The confirmatory portion of the assay involved the vehicle, positive control, and concentration levels of 0.0278, 0.083, 0.278, 0.83, 2.78 ppm AFD1 applied to each tester strain in triplicate. Positive controls without S9 activation were 2-NF for TA98, SA for TA100 and TA1535, 9-AAD for TA1537, and MMS for WP2 *urv*A. The positive control with S9 activation was 2-AA for all strains of bacteria.

Plates were prepared by addition of 0.1 ml bacterial suspension (≥0.3 × 10^9^ cells/ml in late log phase), 0.1 ml of vehicle or test feed ingredient dilution and 0.5 ml of S9 or sham mix were added to 2.0 ml of molten selective top agar at 45 ± 2°C. The test feed ingredient aliquot was replaced by 0.05 ml of the appropriate positive control, when necessary.

All plates were scored using a dissecting microscope to determine the condition of the bacterial background lawn, where a reduction or absence of the lawn indicate test feed ingredient toxicity. Precipitate was visually evaluated following the incubation period. Toxicity and the degree of precipitation were scored relative to the control vehicle-incubated plates.

For each replicate plating, the mean and standard deviation of the number of revertants per plate were calculated and reported. To be considered positive for mutagenicity, the AFD1-containing test feed ingredient must cause an increase in the mean revertants per plate of at least one tester strain over a minimum of two increasing concentrations (≥3-times the mean vehicle control value for strains TA1535 and TA1537, and ≥2-times the mean vehicle control value for strains TA98, TA100, and WP2 *uvr*A, and above the corresponding acceptable vehicle control range for the strains tested). An equivocal response is a biologically relevant increase in revertant count that only partially meets the criteria for a positive response. A response was determined to be negative if it was neither positive nor equivocal.

#### Chromosome Aberration Assay

The *in vitro* mammalian chromosomal aberration assay was used to detect structural chromosome aberrations by exposing HPBL to the AFD1 product as well as concurrent positive and vehicle controls, in the presence and absence of an exogenous metabolic activation system. The assay was performed under GLP in accordance with 21 CFR 58 and followed the Organization for Economic Cooperation and Development (OECD) guidance 473 (11).

The HPBL were treated with test substance, vehicle control, or positive control for 4 h in the absence and presence of S9, and for 20 h in the absence of S9. The vehicle was sterile water (Gibco, MA) and the positive control for tests in the absence of S9 was MMC and CP in the presence of S9. A preliminary toxicity assay was initially conducted, analyzing nine AFD1 concentrations that ranged from 0.0003 to 3 ppm (the limit concentration for this assay). For the preliminary toxicity and the definitive *in vitro* assays, cells were collected ~20 h after initiation (~1.5 normal cell cycles) for analysis during the first division metaphase. Colcemid® was added 2 h prior to cell harvest, at which time the cells were collected, treated with 0.075 M potassium chloride, washed with fixative and slides prepared, and stained with Giemsa. The concentrations evaluated in the definitive assay were 0.75, 1.5, and 2.25 ppm for the non-activated 4-h exposure time point and, 1.5, 2.25, and 3 ppm in the S9-activated 4-h and the non-activated 20-h exposure time points.

The mitotic index was recorded as the percentage of cells in mitosis per 500 cells counted. A minimum of 300 metaphase cellular spreads containing 46 centromeres from each concentration (150 per duplicate) were examined and scored for chromatid-type (e.g., chromatid and isochromatid breaks and exchange figures such as symmetrical and asymmetrical interchanges, triradials, and complex rearrangements) and chromosome-type (i.e., breaks, deletion exchanges, chromosomal disintegrations, and gaps) aberrations. AFD1 would be considered clastogenic if at least one the test concentrations exhibited a statistically significant (*P* ≤ 0.05) and concentration-dependent increase in structural or numerical aberrations when compared with the concurrent negative control, and the results were outside the 95% control limit of the historical negative control data.

#### 26-Week Feline Dietary Study

Personnel feeding the cats, collecting clinical observations, body and food consumption weights, ophthalmology examinations, cat care, socialization, and clinical pathology collection and analysis were blinded to test feed ingredient-related group assignments. The study director, management, study coordinator, and personnel transferring feed into containers were not blinded. The protocol was reviewed and approved by an Institutional Animal Care and Use Committee (IACUC) and a veterinarian was consulted in the overall study design.

Prior to the start of the study, the cats were examined to assure good health and were acclimated for 13 days and generally observed for changes in health and well-being. The cats were then observed twice daily for recorded changes in general appearance or behavior. The cats were randomly placed in rooms (divided by sex) and individually housed. Average room temperature and relative humidity was 18–29°C and 50 ± 20%, respectively, with a 12-h daily photoperiod. Each room maintained a minimum daily average of 10.75 air changes/h. All cats in the study participated in socialization, which included group play in the cat room as well as interactions with technicians and toys. Each assigned group of cats was socialized and exercised separately from other groups.

The cats were randomly allocated to one of four groups, each consisting of five males and five females. The study was a randomized, controlled, blinded study. The study started on Day 0; Group 1 received 39 ppm, Group 2 received 0 ppm, Group 3 received 66 ppm, and Group 4 received 7 ppm AFD1 in the diet; the feeding portion of the study was conducted for 26 weeks. The cats were fed initially according to energy requirements for each cats' bodyweight (based on the feed providing 4,102 kcal energy/kg diet and the metabolic needs of the cats at 59 kcal/kg bodyweight) and this amount was increased or decreased on a weekly basis if a cats' body weight changed by 5% or more relative to starting weight. During the study, it was found that this feeding schedule resulted in weight gain. Therefore, after Day 100 of the study, feeding amounts were provided to each of the cats based on a body condition scoring system [The Nestlé Purina Body Condition System; (12)] using a 1–9 scale completed by the attending veterinarian at least twice per month for the balance of the study, such that the cats maintained a body condition score of 4–6.

Daily observations (including, but not limited to changes in skin, hair, eyes, mucous membranes, respiratory, autonomic, and central nervous function, motor activity, and behavioral pattern) were completed at least once daily beginning on the day of randomization and detailed clinical observations completed by trained technical personnel at least weekly from the day of randomization until the end of the study. Stool consistency was assessed daily. Body weights were determined at least once prior to randomization and approximately weekly until the end of the study. Fresh food was provided once daily, and consumption was quantified daily starting at least 2 weeks prior to the first day of AFD1 feeding; uneaten food was measured in grams at approximately the same time each day. Filtered tap water was provided *ad libitum*. Ophthalmic evaluations were conducted prior to the first day of AFD1 feeding, then at Week 13 and 26.

Blood and urine samples were collected during the study for clinical chemistry, blood coagulation, hematology, and urinalysis. Following an overnight fast, blood was collected (in sodium citrate-coated tubes for coagulation parameters, in K_2_EDTA-coated tubes for hematological parameters, in lithium heparin-coated tubes for taurine analysis, and for clinical chemistry parameters the sample was collected in a clot-activator gel tube) ~7 days prior to the start of the study (but after at least 7 days on control diet), then again on Day 100 (±3 days), and within 1 week of the last day of AFD1 feeding (Day 182). Urine was collected by pan during the daytime period using non-absorbent litter, or when inadequate sample was obtained the non-absorbent litter was left overnight for collection the following morning. Hematological analysis was conducted using the Advia 120 Hematology System, clinical chemistry analysis was conducted using the Advia 1800 Clinical Chemistry System and urinalysis was conducted using the Clinitek Advantus system (Siemens, Malvern, PA). Blood coagulation was analyzed using the Stago STA Compact® Coagulation Analyzer (Diagnostica Stago, Inc., NJ).

The following hematological parameters were evaluated: total red blood cell (RBC) (erythrocyte) count, red cell distribution width (RDW), hemoglobin (HGB), hematocrit (HCT), mean corpuscular volume (MCV), mean corpuscular hemoglobin (MCH), mean corpuscular hemoglobin concentration (MCHC), cytologic morphology, total platelet count, mean platelet volume (MPV), platelet distribution width (PDW), platelets (PLTS), neutrophils (NEU); hemoglobin distribution width (HDW), total white blood cell (WBC; leukocyte) count, lymphocytes (Lymph), monocytes (Mono), eosinophils (Eosin), basophils (Baso), differential blood smear, and reticulocyte (Retic) count (absolute and relative). Prothrombin time (PT), activated partial thromboplastin time (APTT), and thromboplastin time (TT) parameters were analyzed, as well as the following clinical chemistry parameters: glucose, serum urea nitrogen, creatinine (Creat), total protein, albumin (ALB), globulin, albumin/globulin ratio (A/G), total bilirubin, alanine aminotransferase (ALT), sorbitol dehydrogenase (SOD), alkaline phosphatase (ALP), gamma-glutamyl transferase (GGT), aspartate aminotransferase (AST), calcium (Calc), inorganic phosphorus (Phos), sodium (Na+), potassium (K+), chloride (Cl–), and total bile acids. Parameters analyzed from the urine were: clarity, color, specific gravity, blood, ketones, protein, and microscopic examination of sediment, urobilinogen, bilirubin, glucose, pH, leukocytes, and nitrites.

### Statistical Analyses

#### Genotoxicity Studies

Statistical analysis was not conducted for the results of the bacterial reverse mutation assay, as statistical analyses are not required as a part of the OECD 471 (13) protocol guidelines.

For the chromosomal aberration study, a pairwise comparison of the frequency of aberrant cells in each group with the vehicle was statistically analyzed using the Fisher's exact test (*P* ≤ 0.05). The Cochran-Armitage trend test was also used to assess concentration dependent-responsiveness.

#### 26-Week Feline Dietary Study

The test feed ingredient-provided groups were compared to the control group. Mean and standard deviations were calculated for all quantitative data. Continuous group mean data (e.g., food consumption, body weights, clinical pathology) that were examined statistically were evaluated for equality or homogeneity of variance using the Provantis™ Decision Tree statistical structure.

The Decision Tree statistical structure included analysis of variance (ANOVA) and covariance (ACOVA), non-parametric analysis of variance, pairwise tests by the Dunnett's Test for parametric and non-parametric data, simple *t*-Tests, and the Bartlett's Test for homogeneity of variance. A determination of the “best” transformation for each variable was completed for use of either parametric or non-parametric analysis. The use of possible covariates and the homogeneity of means was also determined. The data were then analyzed to test for an AFD1 level-related trend and, if so, the lowest administered AFD1 group affected, based on the Williams Test (parametric data) or the Shirley Test (non-parametric data). If no trend effect was found, but the data showed non-homogeneity of means in the above transformation, then the data were analyzed by a stepwise Dunnett Test (parametric data) or a modified Steel Test (non-parametric data) for evaluation of significant difference from the control group. Any specified pair-wise tests were performed, using the Student *t*-test (parametric) or via non-parametric confidence limits on median differences (non-parametric) between data. In general, statistical tests were performed as two-sided tests with results taken as significant with probability (*P*) levels of < 0.05 or < 0.01, with the exception of trend tests (Williams and Shirley), where only the top level was analyzed using a two-sided test.

## Results and Discussion

### Bacterial Reverse Mutation Assay

The initial toxicity-mutation assay was conducted at 0.00083, 0.00278, 0.0083, 0.0278, 0.083, 0.278, 0.83, and 2.78 ppm AFD1 in water, with the AFD1 forming workable suspensions from 0.75 to 75 ppm and solutions from 0.0225 to 0.225 ppm. Neither precipitate nor toxicity was observed. No positive mutagenic responses were observed with any of the tester strains either in the presence or absence of S9 metabolic activation mix in the initial assay (Table 1).

**Table 1:** Bacterial reverse mutation assay: initial toxicity assay.

			**Revertant colony counts (Mean ± SD)**				
**Metabolic** ** activation**	**Test ingredient**	**Concentration** ** (ppm)**	**TA98**	**TA100**	**TA1535**	**TA1537**	**WP2*uvr*A**
Without activation	Water	100 μL/plate	14 ± 4	81 ± 1	10 ± 0	11 ± 4	23 ± 3
	AFD1	0.00083	18 ± 1	79 ± 1	10 ± 1	8 ± 4	25 ± 9
		0.00278	17 ± 6	83 ± 12	12 ± 3	10 ± 1	27 ± 8
		0.0083	17 ± 2	84 ± 11	8 ± 1	8 ± 7	18 ± 1
		0.0278	19 ± 3	85 ± 1	17 ± 1	10 ± 2	21 ± 6
		0.083	15 ± 2	86 ± 5	12 ± 3	12 ± 2	22 ± 6
		0.278	11 ± 0	88 ± 17	11 ± 3	10 ± 2	21 ± 6
		0.83	11 ± 1	84 ± 8	12 ± 2	12 ± 3	18 ± 5
		2.78	15 ± 0	87 ± 15	13 ± 3	11 ± 5	28 ± 5
	2NF	1.0 μg/plate	89 ± 15				
	SA	1.0 μg/plate		474 ± 52	595 ± 25		
	9AAD	75 μg/plate				719 ± 220	
	MMS	1,000 μg/plate					344 ± 39
With activation	Water	100 μL/plate	22 ± 5	82 ± 10	11 ± 1	12 ± 1	25 ± 13
	AFD1	0.00083	24 ± 4	76 ± 11	12 ± 4	15 ± 6	29 ± 5
		0.00278	20 ± 6	92 ± 6	9 ± 0	10 ± 5	27 ± 6
		0.0083	18 ± 1	89 ± 4	13 ± 2	11 ± 4	24 ± 4
		0.0278	24 ± 4	92 ± 8	11 ± 1	10 ± 0	25 ± 1
		0.083	23 ± 1	86 ± 0	12 ± 4	10 ± 1	26 ± 5
		0.278	22 ± 7	81 ± 4	9 ± 4	9 ± 0	20 ± 3
		0.83	20 ± 6	78 ± 0	9 ± 6	13 ± 3	27 ± 4
		2.78	24 ± 0	90 ± 13	16 ± 2	14 ± 1	29 ± 8
	2AA	1.0 μg/plate	287 ± 33		99 ± 11		
	2AA	2.0 μg/plate		965 ± 52		91 ± 32	
	2AA	15 μg/plate					371 ± 22

*Key to positive control: SA, sodium azide; 2AA, 2-aminoanthracene; 9AAD, 9-Aminoacridine; 2NF, 2-nitrofluorene; MMS, methyl methanesulfonate; SD, standard deviation; AFD1, anti-Fel d 1 IgY*.

Based on the results of the initial assay, the concentrations of the AFD1 in the confirmatory assay were: 0.0278, 0.083, 0.278, 0.83, and 2.78 ppm. As in the initial assay, no precipitate or cytotoxicity was observed. No positive mutagenic responses were observed in any of the tester strains in either the presence or absence of the S9 metabolic activation system (Table 2). Application of the positive controls resulted in the expected increases in revertant colony formation.

**Table 2:** Bacterial reverse mutation assay: confirmatory mutagenicity assay.

			**Revertant colony counts (Mean ± SD)**				
**Metabolic** ** activation**	**Test ingredient**	**Concentration** ** (ppm)**	**TA98**	**TA100**	**TA1535**	**TA1537**	**WP2*uvr*A**
Without activation	Water	100 μL/plate	14 ± 4	87 ± 13	16 ± 1	10 ± 3	31 ± 10
	AFD1	0.0278	17 ± 7	109 ± 14	12 ± 2	6 ± 3	21 ± 5
		0.083	12 ± 3	84 ± 8	20 ± 3	10 ± 5	29 ± 13
		0.278	11 ± 5	115 ± 14	14 ± 3	10 ± 3	30 ± 1
		0.83	19 ± 2	88 ± 18	16 ± 6	10 ± 4	25 ± 5
		2.78	20 ± 4	102 ± 28	14 ± 6	10 ± 1	34 ± 10
	2NF	1.00 μg/plate	113 ± 1				
	SA	1.00 μg/plate		908 ± 126	665 ± 161		
	9AAD	75.0 μg/plate				1,203 ± 70	
	MMS	1,000 μg/plate					383 ± 46
With activation	Water	100 μL/plate	25 ± 4	103 ± 13	12 ± 4	13 ± 2	37 ± 3
	AFD1	0.0278	26 ± 5	112 ± 9	13 ± 2	12 ± 6	38 ± 6
		0.083	28 ± 4	107 ± 16	14 ± 4	8 ± 2	29 ± 3
		0.278	25 ± 6	92 ± 15	9 ± 3	11 ± 4	28 ± 7
		0.83	27 ± 7	97 ± 9	11 ± 3	11 ± 2	31 ± 7
		2.78	25 ± 6	84 ± 23	9 ± 3	11 ± 3	38 ± 4
	2AA	1.00 μg/plate	779 ± 31		230 ± 1		
	2AA	2.00 μg/plate		2,040 ± 141		136 ± 17	
	2AA	15.0 μg/plate					353 ± 34

*Key to positive control: SA, sodium azide; 2AA, 2-aminoanthracene; 9AAD, 9-Aminoacridine; 2NF, 2-nitrofluorene; MMS, methyl methanesulfonate; SD, standard deviation; AFD1, anti-Fel d 1 IgY*.

### Chromosomal Aberration Assay

Cytotoxicity, defined as a ≥50% reduction in mitotic index relative to the vehicle control, was not observed at any concentration in any of the three groups in the preliminary or confirmatory assays. Visible precipitate was observed at the highest concentration (3 ppm AFD1) at the conclusion of the period in the preliminary assay. Based on the lack of cytotoxicity and precipitate formation, the AFD1 concentrations chosen for the chromosome aberration assay were 0.375, 0.75, 1.5, 2.25, and 3 ppm. At the conclusion of the chromosomal aberration period, visible precipitate was seen at concentrations greater or equal to 2.25 ppm in the non-activated 4-h exposure group and at 3 ppm in the S9-activated 4-h and the non-activated 20-h exposure groups.

The results for the positive and vehicle controls indicated that all criteria for a valid assay were met (because the non-activated and S9-activated groups were tested concurrently, the positive control for the non-activated 4-h exposure time point was eliminated). No significant (*P* > 0.05) or concentration-dependent increases in structural or numerical (polyploid or endoreduplicated cells) aberrations were observed in the AFD1-treated groups, with or without S9 metabolic activation (Table 3).

**Table 3:** *In vitro* mammalian chromosome aberration assay in human peripheral blood lymphocytes (HPBL).

**Metabolic activation**	**Test ingredient**	**Concentration (ppm)**	**Cytotoxicity^a^ (% of control)**	**Aberrant cells structural (Mean %)^b^**	**Aberrations per numerical (Mean %)^c^**	**Total cell^b^^,^^d^** ** (Mean ± SD^f^)**	**Polyploid cells** ** (Mean %)^e^**
**20-h continuous**							
Without activation	Water	NA	NA	0.0	0.0	0.000 ± 0.000	0.0
	AFD1	1.5	5	0.0	0.0	0.000 ± 0.000	0.0
	AFD1	2.25	8	0.0	0.0	0.000 ± 0.000	0.0
	AFD1	3 p	9	0.3	0.0	0.003 ± 0.058	0.0
	MMC	0.3 μg/ml	44	23.3^**^	0.0	0.260 ± 0.511	0.0
**4-h with 16 h recovery**							
Without Activation	Water	NA	NA	0.7	0.0	0.007 ± 0.082	0.0
	AFD1	0.75	2	0.0	0.0	0.000 ± 0.000	0.0
	AFD1	1.5	7	0.0	0.0	0.000 ± 0.000	0.0
	AFD1	2.25 p	10	0.0	0.3	0.000 ± 0.000	0.0
**4-h with 16 h recovery**							
With Activation	Water	NA	NA	0.3	0.0	0.003 ± 0.058	0.0
	AFD1	1.5	1	0.0	0.3	0.000 ± 0.000	0.3
	AFD1	2.25	12	0.7	0.0	0.007 ± 0.082	0.0
	AFD1	3 p	7	0.3	0.0	0.003 ± 0.058	0.0
	CP	5 μg/ml	29	14.0^**^	0.0	0.147 ± 0.373	0.0

*MMC, Mitomycin C; CP, Cyclophosphamide; AFD1, Anti-Fel d 1 IgY; NA, Not Applicable; Fisher's Exact Test*,

** *P ≤ 0.01*.

a *Based on mitotic inhibition relative to solvent control*.

b *Does not include cells with only gaps*.

c *Includes polyploid and endoreduplicated cells*.

d *Severely damaged cells counted as 10 aberrations*.

e *Does not include endoreduplicated cell*.

f *SD, Standard Deviation*.

*p, visible precipitate was observed in the medium at the conclusion of the test period*.

### Feline Dietary Study

**Figure 1:**
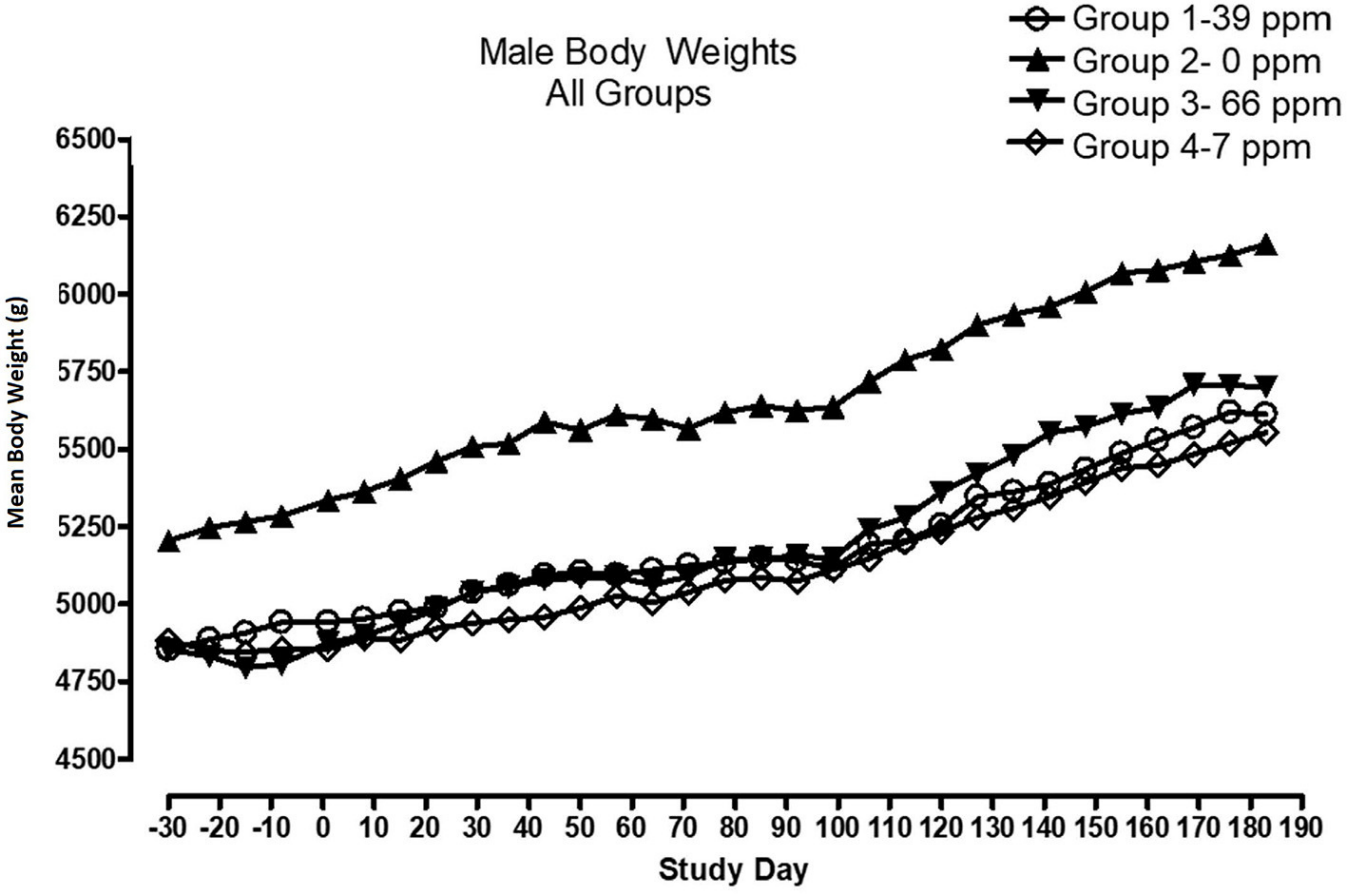
Mean body weights for male cats fed AFD1-enriched dry kibble.

**Figure 2:**
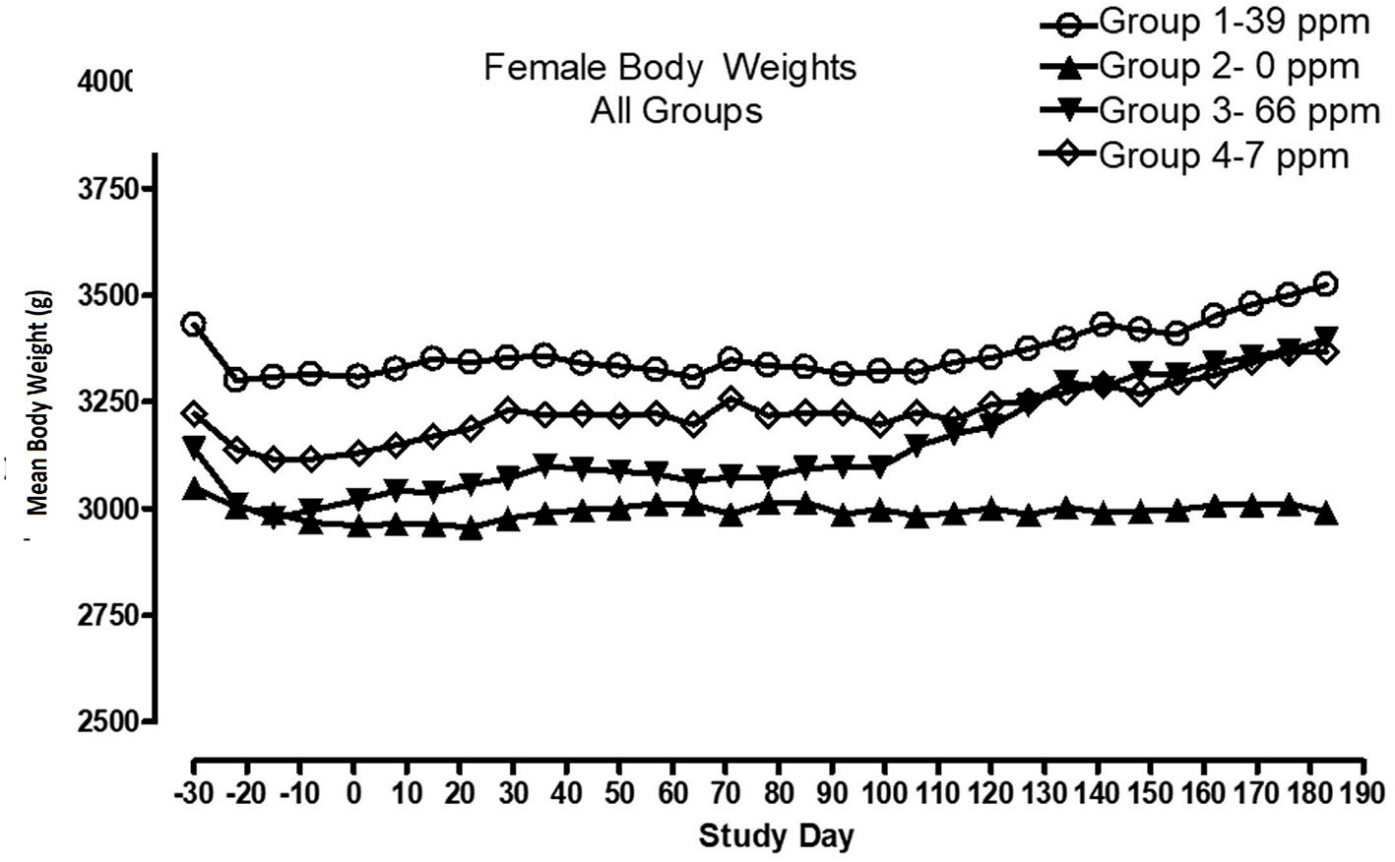
Mean body weights for female cats fed AFD1-enriched dry kibble.

All cats remained in good health through the study. Statistical analysis found that the data for this in-life study were parametric in nature. Data originally provided in Système Internationale (SI) units were converted to conventional units for this publication. There were no significant differences between test and control groups on body weights (Figures 1, 2) or body weight gains (data not shown) in the male cats at the end of the study. Lower mean body weight gains between Days 36 and 43 occurred in the 7 and 66 ppm feed groups, but this was not an AFD1 concentration-dependent response and was only transient in nature, and therefore was not related to consumption of AFD1. There was a statistically significant (*P* < 0.05) increase in mean body weight gain in the female 66 ppm group at the Day 120–127 consumption period, compared to the respective control group (data not shown), but average female body weights in the AFD1-consuming groups were not significantly different from the control group. The mean body weight in the 7 and 39 ppm groups gained 7.85 and 6.38%, respectively, and was within normal biological variation.

**Figure 3:**
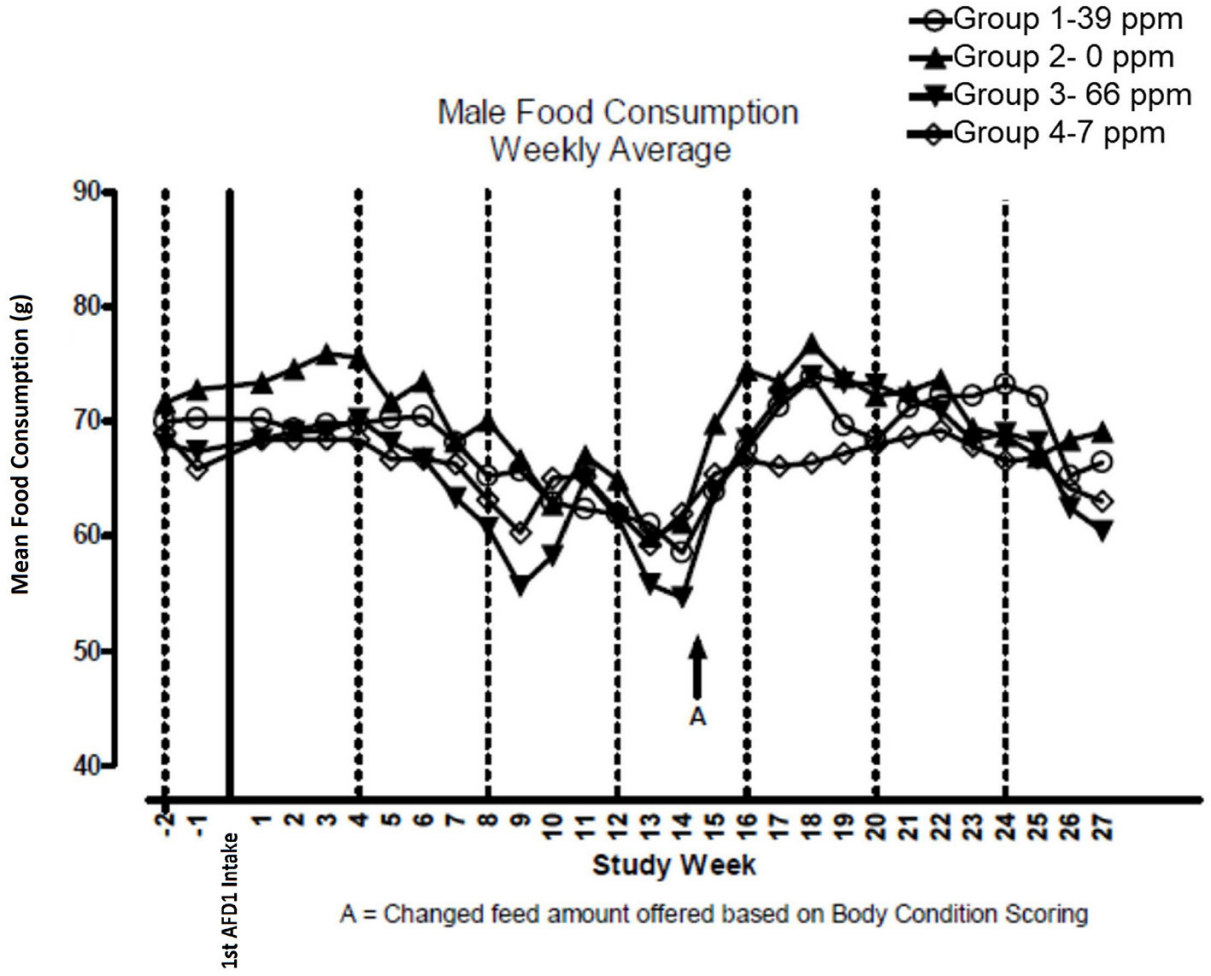
Mean food consumption for male cats fed AFD1-enriched dry kibble.

**Figure 4:**
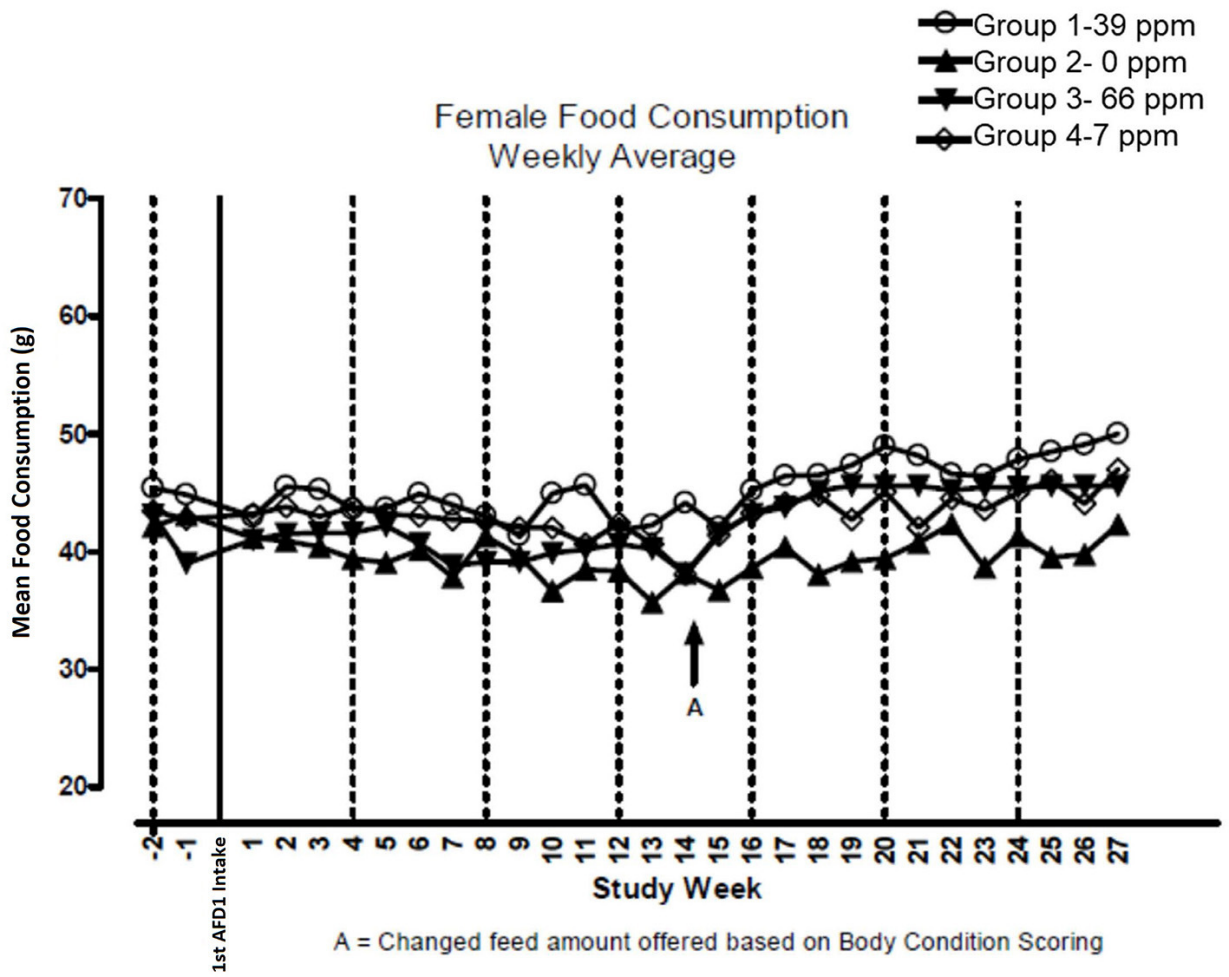
Mean food consumption for female cats fed AFD1-enriched dry kibble.

There were no significant feeding group-dependent differences in consumption of the food containing 7, 39, or 66 ppm AFD1, when compared to the control group (*P* > 0.05) for either male or female cats in the study. The mean average weekly food consumption values are presented in Figure 3 (males) and Figure 4 (females). There were no statistically significant differences between control and test groups in food consumption on any measured day during the study. Prior to Day 100, the amount of food offered each cat was varied each week to attempt to maintain body weight within a 5% range of its Day 1 body weight, while still meeting the metabolic need of 59 kcal/kg body weight (bw). As body weight gain continued even with alteration of the amount of food provided, after Day 100 the feed was adjusted at least weekly according to the Nestlé Purina Body Condition Score [amount of feed given to the cat was increased if the Body Condition Score was below 4 and decreased if the Body Condition Score was above 6 (12)], maintaining metabolic needs of the cats. The male cats consumed an average of 0, 0.09, 0.51, and 0.83 mg AFD1 /kg bw/day; the female cats consumed an average of 0, 0.09, 0.53, and 0.88 mg AFD1/kg bw/day for the 0, 7, 39, and 66 ppm feed groups, respectively, which was less than the 0.11, 0.55, and 1.1 mg/kg bw/day expected for the respective groups.

The clinical pathology data were typical of clinically healthy cats, and generally similar among the groups at each collection interval. There were statistically significant (*P* < 0.05) differences in absolute and percent reticulocytes in the 7 and 66 ppm male feed groups at Day 182 (Table 4) that were not attributed to AFD1 feeding, as there was a lack of a group-response relationship, considerable variation in these parameters prior to AFD1 feeding, the values were within published reference levels [% reticulocyte range: 0.0–0.6%; (14)] and reticulocytes at “10^3^/μL” range: <60 × 10^3^/μL; (14) and the lack of meaningful changes in the mature erythrocyte parameters (i.e., RBC, HGB, or HCT).

**Table 4:** Hematology values (mean ± SD) in male cats prior to and during AFD1 feeding.

**Males**	**Study day**	**AFD1 feed group**				**Reference ranges (14)**
		**0 ppm**	**7 ppm**	**39 ppm**	**66 ppm**	
RBC 10^6^/μL	−71#	9.520 ± 0.497	8.84 ± 0.709	9.200 ± 0.935	10.060 ± 1.358	(5.0–10.0)
	−7	9.870 ± 0.968	10.894 ± 0.688	10.908 ± 1.103	10.934 ± 0.950	
	100	8.756 ± 0.766	9.122 ± 0.956	10.034 ± 1.115	9.582 ± 1.171	
	182	9.112 ± 0.555	9.866 ± 1.147	10.058 ± 0.648	9.766 ± 0.628	
RDW %	−71	15.24 ± 1.01	15.76 ± 0.89	15.18 ± 1.48	15.48 ± 0.35	(15.0–27.0)$
	−7	16.70 ± 1.59	16.60 ± 0.65	17.06 ± 1.77	17.62 ± 1.11	
	100	15.72 ± 0.99	15.48 ± 0.98	16.14 ± 1.52	15.72 ± 0.77	
	182	14.68 ± 0.56	14.72 ± 0.28	15.32 ± 1.22	14.46 ± 0.13	
Hgb g/dL	−71	13.26 ± 1.18	12.02 ± 1.64	12.68 ± 0.56	13.16 ± 1.23	9.8–15.4
	−7	13.38 ± 1.73	13.94 ± 0.84	14.52 ± 0.82	14.10 ± 1.70	
	100	12.84 ± 1.15	12.94 ± 1.25	14.08 ± 0.82	13.72 ± 1.92	
	182	12.76 ± 1.32	13.40 ± 0.94	13.72 ± 0.90	13.30 ± 1.44	
HDW g/dL	−7	1.892 ± 0.148	1.830 ± 0.128	1.934 ± 0.276	1.864 ± 0.074	(1.5–2.8)##
	100	1.946 ± 0.200	1.974 ± 0.192	2.044 ± 0.237	2.006 ± 0.076	
	182	1.732 ± 0.118	1.726 ± 0.161	1.810 ± 0.177	1.788 ± 0.114	
HCT %	−71	40.60 ± 3.05	37.20 ± 4.66	38.60 ± 1.67	40.80 ± 4.21	(30.3–52.3)$
	−7	40.66 ± 5.07	43.94 ± 2.24	45.00 ± 2.79	44.46 ± 5.49	
	100	36.54 ± 3.12	36.86 ± 3.59	40.06 ± 3.34	38.96 ± 5.63	
	182	39.44 ± 3.25	41.42 ± 3.25	41.84 ± 2.99	41.14 ± 4.65	
MCV fL	−71	42.60 ± 1.82	42.00 ± 3.54	42.40 ± 3.65	40.60 ± 2.07	(39–55)
	−7	41.10 ± 1.65	40.38 ± 2.34	41.42 ± 2.51	40.68 ± 3.12	
	100	41.74 ± 1.51	40.52 ± 2.60	40.06 ± 2.04	40.58 ± 1.87	
	182	43.24 ± 1.60	42.18 ± 2.49	41.60 ± 2.17	42.00 ± 2.01	
MCH pg	−71	14.00 ± 0.71	13.40 ± 0.89	13.80 ± 1.48	13.20 ± 0.84	(13–17)
	−7	13.54 ± 0.56	12.82 ± 0.88	13.40 ± 0.93	12.88 ± 0.97	
	100	14.64 ± 0.70	14.26 ± 1.12	14.10 ± 0.97	14.28 ± 0.73	
	182	13.96 ± 0.80	13.68 ± 0.87	13.66 ± 0.86	13.56 ± 0.61	
MCHC g/dL	−71	32.80 ± 0.84	32.40 ± 0.89	32.80 ± 0.84	32.40 ± 0.55	(30–36)
	−7	32.94 ± 0.28	31.72^**^± 0.75	32.30^*^± 0.35	31.76^**^± 0.51	
	100	35.10 ± 0.46	35.16 ± 0.55	35.22 ± 0.99	35.22 ± 0.59	
	182	32.28 ± 0.75	32.40 ± 0.53	32.84 ± 0.66	32.28 ± 0.13	
PLTS 10^3^/μL	−71	335.7 ± 52.5	230.5 ± 17.7	255.5 ± 31.8	338.4 ± 83.7	(300–800)
	−7	346.4 ± 107.6	330.0 ± 157.6	420.2 ± 56.9	449.4 ± 110.9	
	100	324.4 ± 59.0	237.8 ± 117.6	313.2 ± 118.9	412.6 ± 69.4	
	182	313.6 ± 60.7	353.6^*^± 47.2	414.0^*^± 13.0	427.2^**^± 62.5	
Retic Ab 10^3^/μL	−7	20.54 ± 6.97	24.06 ± 8.05	34.08 ± 12.56	37.12^*^± 10.16	(<60)
	100	22.40 ± 4.15	51.02 ± 26.47	40.34 ± 17.31	30.54 ± 15.12	
	182	19.40 ± 6.37	30.18^*^± 4.96	27.44 ± 10.40	32.02^*^± 8.07	
Retic %	−7	0.20 ± 0.07	0.20 ± 0.07	0.30 ± 0.10	0.34^*^± 0.05	(0–0.6)
	100	0.26 ± 0.09	0.56 ± 0.30	0.42 ± 0.16	0.32 ± 0.13	
	182	0.20 ± 0.07	0.32^*^± 0.04	0.28 ± 0.11	0.34^*^± 0.09	
WBC 10^3^/μL	−71	7.70 ± 1.02	9.56 ± 1.83	7.64 ± 2.53	10.14 ± 3.62	(5.5–19.5)
	−7	8.94 ± 1.96	10.02 ± 1.01	11.60 ± 4.37	12.82 ± 4.57	
	100	8.80 ± 2.25	10.76 ± 2.30	11.36 ± 2.76	12.00 ± 4.30	
	182	9.68 ± 2.66	11.62 ± 2.39	12.26 ± 4.73	13.10 ± 3.26	
Neu Ab 10^3^/μL	−71	4.120 ± 0.729	5.940 ± 1.618	4.180 ± 2.899	4.960 ± 1.577	(2.5–12.5)
	−7	4.490 ± 1.560	3.938 ± 1.700	5.014 ± 2.527	5.136 ± 3.177	
	100	3.896 ± 1.128	3.788 ± 1.455	4.212 ± 2.974	3.984 ± 2.185	
	182	4.600 ± 1.314	3.882 ± 1.202	4.160 ± 1.224	4.452 ± 1.361	
Neu %	−7	50.42 ± 12.45	38.42 ± 14.20	41.86 ± 10.88	37.34 ± 13.43	(45–64)
	100	44.08 ± 6.61	34.62 ± 9.20	35.00 ± 16.18	32.48 ± 9.53	
	182	48.26 ± 10.52	33.12^*^± 4.73	35.24^*^± 9.31	33.88^*^± 6.04	
Lymph Ab 10^3^/μL	−71	2.860 ± 1.210	2.820 ± 1.062	2.800 ± 0.718	4.360 ± 1.906	(1.5–7.0)
	−7	3.382 ± 1.344	4.838^*^± 0.912	4.774^*^± 1.127	6.408^**^± 1.385	
	100	3.524 ± 0.544	5.892^*^± 1.284	5.804^*^± 1.877	7.000^**^± 2.551	
	182	3.938 ± 1.583	6.312^*^± 1.501	5.504 ± 1.796	7.452^**^± 2.126	
Lymph %	−7	37.78 ± 11.16	48.62 ± 9.50	43.46 ± 11.28	52.74 ± 13.18	(27–36)
	100	40.88 ± 4.43	54.88^*^± 6.28	52.34^*^± 14.70	58.60^*^± 11.27	
	182	39.88 ± 7.67	54.06^**^± 5.87	45.80 ± 7.85	56.88^**^± 7.50	
Mono Ab 10^3^/μL	−71	0.160 ± 0.055	0.120 ± 0.084	0.10 ± 0.071	0.220 ± 0.164	(0–0.9)
	−7	0.244 ± 0.115	0.234 ± 0.085	0.250 ± 0.084	0.304 ± 0.245	
	100	0.262 ± 0.178	0.244 ± 0.059	0.258 ± 0.041	0.214 ± 0.106	
	182	0.254 ± 0.134	0.260 ± 0.058	0.270 ± 0.075	0.252 ± 0.100	
Mono %	−7	2.78 ± 1.10	2.36 ± 0.86	2.20 ± 0.23	2.20 ± 0.96	(0–5)
	100	2.78 ± 1.14	2.40 ± 0.74	2.32 ± 0.37	1.74^*^± 0.29	
	182	2.64 ± 0.96	2.28 ± 0.66	2.28 ± 0.36	1.92 ± 0.40	
Eosin 10^3^/μL	−71	0.540 ± 0.351	0.640 ± 0.270	0.480 ± 0.045	0.500 ± 0.245	(0–0.8)
	−7	0.778 ± 0.465	0.974 ± 0.624	1.524 ± 1.232	0.930 ± 0.495	
	100	1.092 ± 0.881	0.806 ± 0.274	1.018 ± 0.433	0.786 ± 0.411	
	182	0.880 ± 0.671	1.130 ± 0.164	2.284 ± 2.351	0.930 ± 0.278	
Eosin %	−7	8.68 ± 5.31	10.24 ± 7.71	12.20 ± 4.97	7.36 ± 3.33	(0–4)
	100	11.94 ± 6.82	7.80 ± 3.40	10.08 ± 6.29	6.98 ± 4.25	
	182	8.90 ± 5.17	10.30 ± 3.56	16.34 ± 9.57	7.16 ± 1.91	
Baso Ab 10^3^/μL	−71	0.000 ± 0.000 n	0.000 ± 0.000 n	0.000 ± 0.000 n	0.000 ± 0.000 n	(0–0.2)
	−7	0.016 ± 0.005	0.016 ± 0.005	0.018 ± 0.008	0.022 ± 0.011	
	100	0.010 ± 0.000	0.010 ± 0.000	0.010 ± 0.007	0.006 ± 0.005	
	182	0.010 ± 0.000	0.010 ± 0.000	0.014 ± 0.009	0.014 ± 0.005	
Baso %	−7	0.18 ± 0.15	0.18 ± 0.08	0.16 ± 0.05	0.20 ± 0.07	(0–1)
	100	0.10 ± 0.07	0.12 ± 0.04	0.10 ± 0.00	0.06 ± 0.05	
	182	0.10 ± 0.00	0.10 ± 0.00	0.12 ± 0.08	0.12 ± 0.04	

*AFD1, anti-Fel d 1 IgY; RBC, Red blood cells; RDW, Red cell distribution width; Hgb, Hemoglobin; HDW, Hemoglobin distribution width; HCT, Hematocrit; MCV, Mean corpuscular volume; MCH, Mean corpuscular hemoglobin; MCHC, Mean corpuscular hemoglobin concentration; PLTS, Platelets; Retic, Reticulocytes; WBC, White blood cells; Neu, Neutrophils; Lymph, Lymphocytes; Mono, Monocytes; Eosin, Eosinophils; Baso, Basophils*.

* *Significant at P < 0.05 when compared to the control group within a study day*.

** *Significant at P < 0.01 when compared to the control group within a study day*.

*SD, Standard deviation*.

# *Day−71 data obtained at Vendor location prior to shipment to study site*.

## *Moritz et al. (15)*.

$, IDEXX ProCyte Dx* Hematology Analyzer (16).

*n, Data not appropriate for statistical analysis*.

Platelet counts varied considerably among the groups at each collection interval, as is typical of clinically healthy cats. Trumel et al. (17) found the coefficient of variation (CV, %) to be 14.8% for intraindividual and 18.9% for interindividual variability for a set of 14 cats during a three-month study. The significantly higher mean platelet counts observed in the male groups on Day 182 (Table 4) were not attributed to AFD1 because of the lack of a clear AFD1 level response and the fact that females were not similarly affected (Table 5). One control male was inappetent and hypoactive on Day 157 and had a lower platelet count (121 × 10^3^/μL /μL) with no MPV value obtained and no platelet clumps observed in the blood smear. The cat was hypoactive again (but not inappetent) on Days 158–160, but no abnormalities were found for the remainder of the study for this cat. The platelet count for this cat was also slightly lower (193 × 10^3^/μL) on Day−7 but >300 × 10^3^/μL on Days 100 and 182. This control male recovered while still on study with continued feeding of control diet and had unremarkable clinical pathology values on Day 182.

**Table 5:** Hematology values (mean ± SD) in female cats prior to and during AFD1 feeding.

**Females**	**Study day**	**AFD1 level**				**Reference ranges (14)**
		**0 ppm**	**7 ppm**	**39 ppm**	**66 ppm**	
RBC 10^6^/μL	−71#	8.800 ± 0.985	8.400 ± 0.762	8.440 ± 0.838	8.420 ± 0.920	(5.0–10.0)
	−7	9.926 ± 0.691	9.828 ± 0.280	9.548 ± 1.001	9.624 ± 0.760	
	100	8.660 ± 0.748	9.028 ± 0.396	9.294 ± 0.864	7.680^*^± 0.656	
	182	9.524 ± 0.493	9.258 ± 0.474	8.916 ± 1.271	8.482 ± 1.152	
RDW %	−71	15.00 ± 0.71	15.45 ± 1.11	14.96 ± 0.77	14.66 ± 0.34	(15.0–27.0)$
	−7	15.78 ± 0.82	15.64 ± 0.92	14.34 ± 0.42	15.28 ± 1.01	
	100	15.70 ± 0.83	15.34 ± 0.77	15.08 ± 0.86	15.36 ± 1.78	
	182	15.06 ± 0.93	14.46 ± 0.96	14.58 ± 0.91	14.22 ± 0.38	
Hgb g/dL	−71	12.66 ± 1.53	12.14 ± 1.15	12.06 ± 0.66	12.30 ± 1.42	(9.8–15.4)
	−7	13.36 ± 1.05	13.14 ± 1.32	12.94 ± 1.67	13.48 ± 0.58	
	100	12.48 ± 0.26	13.44 ± 0.80	13.96^*^± 1.16	11.50 ± 0.94	
	182	13.36 ± 0.45	12.42 ± 2.11	12.66 ± 1.15	12.32 ± 1.32	
HDW g/dL	−7	1.862 ± 0.174	1.832 ± 0.104	1.750 ± 0.102	1.664^*^± 0.110	(1.5–2.8)##
	100	1.920 ± 0.150	1.926 ± 0.069	1.956 ± 0.185	1.836 ± 0.066	
	182	1.802 ± 0.140	1.760 ± 0.102	1.756 ± 0.143	1.624^*^± 0.093	
HCT %	−71	39.20 ± 4.76	36.75 ± 3.40	36.80 ± 1.64	37.60 ± 4.34	(30.3–52.3)$
	−7	41.92 ± 2.85	42.80 ± 2.70	39.40 ± 4.86	42.38 ± 2.11	
	100	35.96 ± 1.81	38.24 ± 2.26	40.10 ± 4.18	32.24 ± 2.51	
	182	41.48 ± 1.07	40.58 ± 3.54	38.66 ± 33.30	37.46 ± 4.45	
MCV fL	−71	44.60 ± 1.82	43.75 ± 2.22	43.80 ± 3.35	44.20 ± 1.92	(39–55)
	−7	42.22 ± 1.61	43.54 ± 2.81	41.40 ± 4.13	44.14 ± 1.94	
	100	41.64 ± 1.59	42.36 ± 2.60	43.36 ± 5.05	42.08 ± 2.03	
	182	43.60 ± 2.06	43.82 ± 2.76	43.74 ± 4.22	44.30 ± 2.00	
MCH pg	−71	14.20 ± 0.84	14.00 ± 0.82	14.40 ± 1.34	14.60 ± 0.89	(13–17)
	−7	13.46 ± 0.93	13.36 ± 1.13	13.60 ± 1.56	14.06 ± 0.86	
	100	14.46 ± 0.98	14.86 ± 0.92	15.12 ± 1.56	14.98 ± 0.93	
	182	14.10 ± 1.07	13.40 ± 1.91	14.36 ± 1.52	14.60 ± 0.88	
MCHC g/dL	−71	32.20 ± 0.84	32.50 ± 0.58	33.00 ± 0.71	32.60 ± 0.55	(30–36)
	−7	31.82 ± 1.34	30.68 ± 1.86	32.82 ± 0.48	31.82 ± 0.66	
	100	34.70 ± 1.19	35.08 ± 0.37	34.88 ± 0.88	35.60 ± 0.64	
	182	32.24 ± 1.18	30.50 ± 3.48	32.76 ± 0.55	32.92 ± 0.55	
PLTS 10^3^/μL	−71	292.5 ± 89.4	356.0 ± 141.5	248.8 ± 43.6	373.3 ± 54.5	(300–800)
	−7	364.8 ± 162.2	362.2 ± 101.2	398.0 ± 61.5	373.2 ± 41.2	
	100	292.8 ± 126.7	393.8 ± 114.5	312.4 ± 177.1	355.8 ± 62.8	
	182	360.6 ± 157.2	412.8 ± 88.0	420.5 ± 51.1	380.2 ± 38.8	
Retic Ab 10^9^/L	−7	26.04 ± 12.73	23.20 ± 14.97	16.72 ± 5.24	22.30 ± 8.79	(<60)
	100	39.30 ± 14.06	34.00 ± 24.66	30.62 ± 9.25	23.54 ± 6.20	
	182	19.42 ± 8.06	16.38 ± 9.03	16.32 ± 4.74	18.00 ± 5.99	
Retic %	−7	0.28 ± 0.13	0.24 ± 0.15	0.16 ± 0.05	0.24 ± 0.09	(0–0.6)
	100	0.44 ± 0.15	0.40 ± 0.25	0.36 ± 0.13	0.32 ± 0.08	
	182	0.20 ± 0.10	0.18 ± 0.08	0.18 ± 0.04	0.24 ± 0.05	
WBC 10^3^/μL	−71	8.16 ± 1.51	7.96 ± 3.56	8.82 ± 1.54	6.92 ± 2.32	(5.5–19.5)
	−7	11.92 ± 3.28	8.86^*^± 2.57	9.02^*^± 1.44	8.26^*^± 2.11	
	100	10.14 ± 1.06	7.84 ± 2.48	9.72 ± 3.63	6.86 ± 1.98	
	182	10.38 ± 1.89	7.86 ± 1.53	8.44 ± 1.90	10.06 ± 2.70	
Neu Ab 10^3^/μL	−71	4.800 ± 1.463	4.820 ± 2.520	5.040 ± 1.905	3.540 ± 0.904	(2.5–12.5)
	−7	5.876 ± 2.938	3.910 ± 1.696	3.856 ± 0.786	3.214 ± 1.527	
	100	4.384 ± 1.336	3.190 ± 1.599	3.474 ± 2.316	2.486 ± 0.759	
	182	4.940 ± 1.514	3.618 ± 0.924	3.432 ± 0.708	3.946 ± 1.583	
Neu %	−7	47.24 ± 10.18	44.18 ± 14.34	42.78 ± 6.55	38.70 ± 13.14	(45–64)
	100	43.10 ± 11.38	39.56 ± 7.87	34.68 ± 11.15	36.16 ± 5.99	
	182	46.98 ± 7.15	46.06 ± 5.59	42.58 ± 12.98	39.00 ± 9.00	
Lymph Ab 10^3^/μL	−71	1.900 ± 0.515	2.020 ± 0.861	2.780 ± 1.110	2.420 ± 0.823	(1.5–7.0)
	−7	4.322 ± 0.805	3.506 ± 1.711	3.950 ± 0.948	3.780 ± 1.495	
	100	4.180 ± 1.270	3.220 ± 0.920	4.830 ± 1.997	3.282 ± 1.141	
	182	3.402 ± 0.944	2.716 ± 0.795	3.358 ± 1.489	4.276 ± 1.695	
Lymph %	−7	36.86 ± 3.59	38.64 ± 10.55	43.66 ± 6.01	45.76 ± 11.74	(27–36)
	100	41.68 ± 13.10	41.70 ± 6.27	49.96 ± 12.34	47.38 ± 4.37	
	182	33.54 ± 10.36	34.28 ± 5.79	38.18 ± 11.39	42.38 ± 10.70	
Mono Ab 10^3^/μL	−71	0.240 ± 0.55	0.180 ± 0.130	0.220 ± 0.130	0.180 ± 0.084	(0–0.9)
	−7	0.324 ± 0.076	0.326 ± 0.121	0.244 ± 0.078	0.270 ± 0.137	
	100	0.326 ± 0.213	0.274 ± 0.084	0.312 ± 0.124	0.222 ± 0.115	
	182	0.334 ± 0.110	0.276 ± 0.061	0.226 ± 0.071	0.268 ± 0.061	
Mono %	−7	2.78 ± 0.63	3.78 ± 1.18	2.66 ± 0.46	3.26 ± 1.30	(0–5)
	100	3.12 ± 1.76	3.78 ± 1.50	3.36 ± 1.13	3.42 ± 1.60	
	182	3.20 ± 0.75	3.68 ± 1.21	2.70 ± 0.60	2.96 ± 1.44	
Eosin Ab 10^3^/μL	−71	1.180 ± 0.722	0.900 ± 0.636	0.780 ± 0.356	0.740 ± 0.598	(0–0.8)
	−7	1.364 ± 0.532	1.070 ± 0.526	0.940 ± 0.196	0.946 ± 0.301	
	100	1.210 ± 0.494	1.114 ± 0.613	1.068 ± 0.354	0.834 ± 0.215	
	182	1.650 ± 0.547	1.200 ± 0.207	1.396 ± 1.219	1.556 ± 1.052	
Baso Ab 10^3^/μL	−71	0.000 ± 0.000 n	0.000 ± 0.000 n	0.000 ± 0.000 n	0.000 ± 0.000 n	(0–0.2)
	−7	0.022 ± 0.016	0.016 ± 0.009	0.012 ± 0.004	0.024 ± 0.011	
	100	0.008 ± 0.008	0.006 ± 0.005	0.010 ± 0.007	0.008 ± 0.004	
	182	0.020 ± 0.029	0.008 ± 0.004	0.004 ± 0.005	0.006 ± 0.005	
Baso %	−7	0.18 ± 0.08	0.18 ± 0.08	0.12 ± 0.04	0.32 ± 0.16	(0–1)
	100	0.08 ± 0.04	0.08 ± 0.04	0.10 ± 0.07	0.14 ± 0.05	
	182	0.24 ± 0.38	0.12 ± 0.04	0.04 ± 0.05	0.10 ± 0.07	

*AFD1, anti-Fel d 1 IgY; RBC, Red blood cells; RDW, Red cell distribution width; Hgb, Hemoglobin; HDW, Hemoglobin distribution width; HCT, Hematocrit; MCV, Mean corpuscular volume; MCH, Mean corpuscular hemoglobin; MCHC, Mean corpuscular hemoglobin concentration; PLTS, Platelets; Retic, Reticulocytes; WBC, White blood cells; Neu, Neutrophils; Lymph, Lymphocytes; Mono, Monocytes; Eosin, Eosinophils; Baso, Basophils*.

* *Significant at P < 0.05 when compared to the control group within a study day*.

SD, Standard deviation.

# *Day−71 data obtained at Vendor location prior to shipment to study site*.

## *Moritz et al. (15)*.

$ IDEXX ProCyte Dx* Hematology Analyzer (16).

*n, Data not appropriate for statistical analysis*.

Obtaining accurate platelet counts in cats is challenging as clumping, a common event in individual cats, causes spuriously lower counts (18). Additionally, platelet clumps are not always apparent on the blood smear, but the wide variation in MPV in this study (~9–20 fL) indicated the wide range of platelet size and/or presence of platelet clumps. The differences in platelet counts in this study were attributed to biologic variability of clinically healthy cats (17).

The statistically significant increase in HBG in the 39 ppm female group on Day 100 was not attributed to AFD1 because of the lack of an AFD1-related response effect, and the lack of concurrent changes in either RBC count or HCT levels (Table 5), and was well within the HBG range (~9.8–15.4) reported by Merck (14). The lower (*P* < 0.05) mean HDW in the 66 ppm female group on Day 182 was not attributed to AFD1 nor adverse in nature, as the mean HDW value on Day 182 was virtually unchanged from the value at acclimation Day−7 and therefore an indicator of normal biological variation, and was within the published reference interval of 1.4–2.0 g/dL [140–200 g/L; (19)].

The significantly higher mean absolute lymphocyte counts observed in all male groups provided AFD1 on Day 100 and in the 7 ppm and 66 ppm male groups on Day 182 had very wide standard deviations and were typical of physiologic (nervous) excitement with release of epinephrine resulting in splenic contraction followed by the release of lymphocytes into the circulation (20). This commonly occurs in cats, and the counts were neither pathologic nor clearly AFD1 level dependent; Trumel et al. (17) reported absolute lymphocyte levels that varied from ~1.0 to almost 7.0 × 10^3^/μL with a reference interval of >10.0, while Merck (14) states an absolute lymphocyte reference range of 1.5–7.0 (× 10^3^/μL). Similar significant elevations were not observed in the female groups when compared with the respective control group at any interval. The increase in absolute numbers of lymphocytes resulted in concurrent significant increases in the percent lymphocyte values, which were higher than publicly available reference range [% lymphocytes: 27–36%; (14)]. Although there were significant decreases in the percentage of neutrophils in all treated male groups on Day 182, the lack of an AFD1 level-based response, and lack of concurrent changes in the mean absolute neutrophil counts indicates the statistical changes were not due to consumption of the test substance and were not adverse in nature. Similarly, the lack of significant changes in the mean absolute monocyte counts and lack of any level-related responsiveness indicates the significant decrease in the percent monocytes in the 66 ppm group was not AFD1-related and not adverse. The percent monocyte values were also within publicly available reference ranges [0–5%; (14)]. Cytological analysis found no abnormal morphology findings (data not shown).

Statistically significant differences observed in the mean values for TT varied in effect (increased or decreased), with higher values in 7 ppm and 66 ppm male and female groups (Table 6), and the 39 ppm male group on Day 100, and lower values for the 7, 39, and 66 ppm female groups on Day 182. The changes in TT values were not consistently increasing or decreasing with increasing AFD1 consumption (either over time or in amount) and were not consistent with decreases seen in APTT in the female AFD1 groups or significant decreases in liver function (as discussed below). In addition, there was a lack of a clear AFD1-related response and most mean TT values in the AFD1 groups varied <2 s from those of the control group; therefore, these effects were not pathologic in nature and were not considered related to AFD1 ingredient consumption. Statistically significant differences observed in the mean values for PT in 7, 39, and 66 ppm female groups on Day 100 were not attributed to AFD1 because of the lack of an AFD1-related response and were not seen on Day 182 after continuous feeding of AFD1 and the values fell within publicly available reference ranges [PT: 10.0–15.3 s; (21)]. Similar to APTT [reference range at 11.2–16.0 s; (21)], the mean PT value in control females on Day 100 was greater than the mean value from other groups at any interval, further indicating that slightly greater control group value increased the chance that the values found in the AFD1-consuming groups would be statistically lower than the control value.

**Table 6:** Analysis of the coagulation of male and female cat blood fed AFD1 (mean ± SD).

	**Study day**	**AFD1 level**				**Reference ranges**
		**0 ppm**	**7 ppm**	**39 ppm**	**66 ppm**	
**Males**						
APTT (s)	−7	11.84 ± 1.12	11.86 ± 0.92	12.14 ± 0.87	11.28 ± 0.77	11.2–16.0#
	100	12.48 ± 1.56	11.74 ± 0.82	12.00 ± 0.97	11.66 ± 0.73	
	182	11.88 ± 0.49	12.36 ± 0.63	12.02 ± 0.56	11.96 ± 0.55	
PT (s)	−7	11.14 ± 0.47	11.06 ± 0.57	11.48 ± 0.54	11.52 ± 0.52	10.0–15.3#
	100	12.18 ± 0.68	11.46 ± 0.83	12.00 ± 0.73	11.34 ± 0.56	
	182	11.18 ± 0.29	11.24 ± 0.70	11.48 ± 0.36	11.46 ± 0.63	
TT (s)	−7	16.20 ± 0.92	16.62 ± 0.57	15.66 ± 0.52	16.46 ± 1.01	13.4–19.1##
	100	15.76 ± 0.80	17.30^**^± 1.02	17.42^**^± 0.93	17.44^**^± 0.56	
	182	17.26 ± 1.23	17.46 ± 0.68	17.06 ± 0.98	17.42 ± 0.99	
**Females**						
APTT (s)	−7	11.70 ± 1.38	11.72 ± 0.71	11.56 ± 0.68	11.94 ± 0.67	11.2–16.0#
	100	13.76 ± 1.23	12.16^**^± 0.52	11.70^**^± 0.41	12.40^**^± 0.92	
	182	12.38 ± 0.24	12.48 ± 0.75	12.24 ± 0.18	12.22 ± 1.01	
PT (s)	−7	12.26 ± 0.82	12.20 ± 1.75	11.76 ± 0.71	12.08 ± 0.75	10.0–15.3#
	100	13.08 ± 0.36	11.86^*^± 0.61	12.32^*^± 0.76	12.24^*^± 0.87	
	182	11.78 ± 0.89	12.04 ± 0.62	12.08 ± 0.41	11.94 ± 0.87	
TT (s)	−7	16.66 ± 1.34	17.22 ± 1.36	15.92 ± 1.08	15.68 ± 0.61	13.4–19.1##
	100	16.06 ± 0.70	18.26^*^± 0.83	16.36 ± 0.63	16.30^*^± 0.99	
	182	18.12 ± 0.70	17.84^*^± 0.67	16.82^*^± 0.44	16.56^**^± 0.59	

*AFD1, anti-Fel d 1 IgY; APTT, Activated Partial Thromboplastin Time; PT, Prothrombin Time; TT, Thrombin Time*.

* *Significant at P < 0.05 when compared to the control group within a study day*;

** Significant at P < 0.01 when compared to the control group within a study day;

# *UC Davis (21)*;

## *Engelen et al. (22)*.

*SD, Standard deviation*.

The significant differences in hematologic values between the AFD1-treatment groups and the control group did not follow a specific treatment-related progression, were not consistent between the male and female groups and were within publicly available reference ranges. There were significant differences in reticulocyte concentrations between the 7 and 66 ppm male AFD1 groups, compared to the control group, but this effect was not concentration-dependent, was not seen in the female groups, and was not reflective of any changes in the mature erythrocyte parameters (i.e., RBC, HGB, or HCT), with no resulting decreases in lymphocyte counts that would indicate an inability of reticulocyte maturation/differentiation. The higher platelet counts found in the male groups on Day 182 was not consistent with effects seen in the female groups and varied considerably; a clear parallel relationship between changes in platelet counts and RDW, MCV, or HCT were not found, an effect that may occur during a toxicological response for any of these parameters (23).

Clinical chemistry data is provided in Table 7 (males) and Table 8 (females). Minimal to mild hemolysis was observed in the serum of many cats, but there was no apparent effect on the chemistry results. SDH was not measured in 66 ppm male group Cat 22 on Day 182 because of insufficient sample, and bile acids were not measured in 66 ppm group female Cat 30 on Day 100 (inadvertently not run). The serum chemistry data were generally similar among the groups, with a few noteworthy observations described below. In general, all measured values were within the ranges expected for clinically healthy cats (24). No effect of dietary AFD1 was found on whole blood taurine levels in the male cats (Table 7), with no AFD1-dependent differences at any interval, when compared to the respective control group (*P* > 0.05).

**Table 7:** Clinical chemistry values (mean ± SD) in male cats prior to and during AFD1 feeding.

**Males**	**Study day**	**AFD1 level**				**Reference ranges (14)**
		**0 ppm**	**7 ppm**	**39 ppm**	**66 ppm**	
Glucose mg/dL	−70#	77.2 ± 7.5	76.0 ± 5.2	73.8 ± 6.9	78.0 ± 7.7	60–120
	−7	72.6 ± 8.4	77.0 ± 4.1	71.4 ± 7.9	91.8^*^± 18.9	
	100	73.8 ± 6.2	75.6 ± 5.2	75.2 ± 11.4	82.6 ± 13.9	
	182	69.2 ± 4.1	71.4 ± 2.3	77.0 ± 18.6	78.6 ± 9.5	
Serum urea nitrogen mg/dL	−70	27.8 ± 1.9	27.0 ± 3.5	24.0 ± 3.9	24.6 ± 3.8	19–34
	−7	24.2 ± 1.1	24.4 ± 3.4	23.4 ± 1.1	23.4 ± 3.0	
	100	28.6 ± 3.9	29.0 ± 2.3	29.0 ± 2.3	24.8 ± 3.0	
	182	26.4 ± 3.6	25.2 ± 3.6	26.2 ± 3.0	24.2 ± 2.9	
Creat mg/dL	−70	1.40 ± 0.16	1.46 ± 0.18	1.30 ± 0.16	1.50 ± 0.14	0.9–2.2
	−7	1.24 ± 0.15	1.40 ± 0.19	1.28 ± 0.15	1.44 ± 0.11	
	100	1.28 ± 0.08	1.44^**^± 0.13	1.62^**^± 0.16	1.60^**^± 0.14	
	182	1.36 ± 0.15	1.24 ± 0.11	1.30 ± 0.12	1.38 ± 0.18	
Total bilirubin mg/dL	−70	0.00 ± 0.00	0.02 ± 0.04	0.00 ± 0.00	0.02 ± 0.04	0–0.1
	−7	0.00 ± 0.00	0.00 ± 0.00	0.00 ± 0.00	0.02 ± 0.04	
	100	0.00 ± 0.00 n	0.00 ± 0.00 n	0.00 ± 0.00 n	0.00 ± 0.00 n	
	182	0.00 ± 0.00 n	0.00 ± 0.00 n	0.00 ± 0.00 n	0.00 ± 0.00 n	
Bile acids μg/mL	−7	0.028 ± 0.040	0.000 ± 0.000	0.023 ± 0.037	0.533 ± 0.770	0–2.04$
	100	1.360 ± 1.547	0.000^*^± 0.000	0.255 ± 0.311	0.349^*^± 0.407	
	182	0.245 ± 0.405	0.207 ± 0.108	0.209 ± 0.080	0.317 ± 0.220	
AST U/L	−70	25.6 ± 3.5	25.2 ± 7.8	26.8 ± 6.1	22.4 ± 3.4	7–38
	−7	32.0 ± 8.6	28.2 ± 5.2	28.6 ± 3.3	38.4 ± 12.6	
	100	26.8 ± 2.6	26.8 ± 5.7	31.6 ± 3.8	29.4 ± 4.2	
	182	28.0 ± 3.8	25.2 ± 3.3	29.8 ± 5.0	27.4 ± 4.6	
ALT U/L	−70	75.2 ± 7.4	79.6 ± 36.9	95.0 ± 12.4	75.8 ± 31.3	25–97
	−7	47.4 ± 4.8	57.8^*^± 11.8	61.2^*^± 5.9	61.8^*^± 7.4	
	100	47.4 ± 8.7	52.4 ± 8.7	63.4 ± 12.5	73.2^*^± 25.1	
	182	52.8 ± 9.3	51.4 ± 6.7	61.8 ± 8.1	70.8 ± 25.2	
ALP U/L	−70	59.8 ± 13.8	49.4 ± 17.8	61.8 ± 14.2	35.4 ± 5.2^*^	0–45
	−7	30.4 ± 13.1	33.4 ± 11.7	52.4^*^± 13.2	26.8 ± 4.7	
	100	36.8 ± 8.9	32.8 ± 5.6	49.2 ± 16.9	26.6 ± 3.8	
	182	30.8 ± 7.9	28.4 ± 5.1	34.2 ± 10.2	26.4 ± 4.0	
SDH U/L	−7	0.94 ± 0.82	2.08 ± 1.95	3.34 ± 2.28	3.48 ± 2.90	3.9–7.7$
	100	0.00 ± 0.00	0.00 ± 0.00	0.24 ± 0.54	0.06 ± 0.13	
	182	3.76 ± 1.08	2.80 ± 0.45	7.26^*^± 3.72	3.23 ± 0.99	
ALB g/dL	−70	3.64 ± 0.27	3.64 ± 0.26	3.76 ± 0.25	3.70 ± 0.12	2.8–3.9
	−7	3.14 ± 0.34	3.46^*^± 0.09	3.54^*^± 0.19	3.52^*^± 0.22	
	100	3.42 ± 0.24	3.42 ± 0.16	3.70 ± 0.29	3.64 ± 0.30	
	182	3.22 ± 0.33	3.30 ± 0.12	3.54 ± 0.23	3.50 ± 0.31	
A/G	−70	1.18 ± 0.19	1.10 ± 0.14	1.28 ± 0.19	1.22 ± 0.11	0.45–1.45$
	−7	0.88 ± 0.11	0.90 ± 0.07	1.04^*^± 0.09	0.92 ± 0.04	
	100	0.90 ± 0.14	0.94 ± 0.05	1.10^*^± 0.12	1.00 ± 0.07	
	182	0.98 ± 0.16	1.02 ± 0.04	1.14 ± 0.11	1.12 ± 0.04	
Total protein g/dL	−70	6.76 ± 0.36	6.96 ± 0.25	6.76 ± 0.60	6.80 ± 0.30	6.0–7.9
	−7	6.66 ± 0.72	7.48 ± 0.25	6.94 ± 0.47	7.34^*^± 0.54	
	100	7.22 ± 0.31	7.06 ± 0.36	7.12 ± 0.75	7.28 ± 0.74	
	182	6.46 ± 0.38	6.58 ± 0.15	6.70 ± 0.59	6.66 ± 0.74	
Na^+^ mEq/L	−70	153.8 ± 1.6	153.4 ± 1.7	153.6 ± 0.9	152.0 ± 1.2	146–156
	−7	140.8 ± 4.3	153.4^**^± 2.2	147.2^*^± 6.6	155.2^**^± 3.8	
	100	152.6 ± 1.5	152.4 ± 0.9	153.0 ± 3.5	154.0 ± 4.5	
	182	145.2 ± 3.6	143.8 ± 2.2	146.8 ± 6.0	149.6 ± 4.3	
K^+^ mEq/L	−70	4.46 ± 0.32	4.66 ± 0.21	4.18 ± 0.28	4.62 ± 0.19	3.7–6.1
	−7	4.48 ± 0.42	5.46^**^± 0.29	4.96^*^± 0.45	5.32*± 0.38	
	100	5.16 ± 0.26	5.10 ± 0.20	5.42 ± 0.54	5.54 ± 0.98	
	182	4.98 ± 0.23	4.96 ± 0.53	5.02 ± 0.72	5.38 ± 0.24	
Calc mg/dL	−70	9.66 ± 0.21	9.88 ± 0.41	10.10 ± 0.48	9.64 ± 0.27	8.7–11.7
	−7	9.02 ± 0.41	9.94^**^± 0.34	9.78^**^± 0.33	9.80^**^± 0.29	
	100	9.70 ± 0.24	9.68 ± 0.33	10.06 ± 0.94	10.02 ± 0.49	
	182	9.14 ± 0.36	9.28 ± 0.36	9.54 ± 0.55	9.68 ± 0.40	
Phos mg/dL	−7	4.68 ± 0.11	5.76^**^± 0.59	5.94^**^± 0.48	6.06^**^± 0.68	3.0–6.1
	100	5.34 ± 0.19	4.64 ± 0.38	5.20 ± 0.60	5.00 ± 0.91	
	182	4.76 ± 0.36	4.42 ± 0.26	5.12 ± 0.68	5.02 ± 0.68	
Cl^−^ mEq/L	−70	115.2 ± 0.8	114.4 ± 2.3	115.0 ± 2.3	114.0 ± 0.7	115–130
	−7	110.6 ± 3.0	120.2^**^± 1.9	114.4 ± 6.3	121.8^**^± 2.6	
	100	122.0 ± 2.1	121.0 ± 1.0	120.4 ± 1.1	121.2 ± 3.6	
	182	115.4 ± 2.5	114.6 ± 1.5	116.8 ± 6.1	119.0 ± 2.7	
Blood taurine nmol/mL	−7/−6	440.2 ± 37.0	433.2 ± 51.1	467.4 ± 91.1	507.6 ± 113.5	275–701$$
	100/101	324.6 ± 60.8	265.2 ± 60.3	271.2 ± 69.1	374.0 ± 35.5	
	182	376.4 ± 31.0	392.4 ± 37.0	387.2 ± 124.7	366.6 ± 60.6	

*AFD1, anti-Fel d 1 IgY; Creat, Creatinine; AST, Aspartate transferase; ALT, Alanine transferase; ALP, Alkaline phosphatase; SDH, Sorbitol Dehydrogenase; ALB, Albumin; A/G, Albumin/Globulin Ratio; Na^+^, Sodium; K^+^, Potassium; Calc, Calcium; Cl^−^, Chloride; Phos, Phosphorus*.

* *Significant at P < 0.05 when compared to the control group within a study day*.

** *Significant at P < 0.01 when compared to the control group within a study day*.

# *Day−70 data obtained at Vendor location prior to shipment to study site*;

$ *Kaneko et al. (24)*;

$$ *UC Davis (25)*.

*SD, Standard deviation [38]*.

*n, Data not appropriate for statistical analysis*.

**Table 8:** Clinical chemistry values (mean ± SD) in female cats prior to and during AFD1 feeding.

**Females**	**Study day**	**AFD1 level**				**Reference ranges (14)**
		**0 ppm**	**7 ppm**	**39 ppm**	**66 ppm**	
Glucose mg/dL	−70	73.2 ± 3.9	72.0 ± 1.6	76.8 ± 5.9	77.0 ± 2.5	60–120
	−7	102.8 ± 25.1	90.4 ± 25.6	66.4 ± 4.0	92.0 ± 22.5	
	100	83.8 ± 19.1	73.8 ± 3.3	85.4 ± 25.4	83.2 ± 17.1	
	182	76.2 ± 25.4	80.0 ± 13.5	70.4 ± 11.2	74.2 ± 9.4	
Serum urea nitrogen mg/dL	−70	24.6 ± 4.8	25.6 ± 1.3	25.2 ± 2.2	25.8 ± 4.2	19–34
	−7	21.4 ± 2.4	24.0 ± 2.7	22.8 ± 0.8	21.0 ± 2.9	
	100	25.0 ± 2.6	27.6 ± 1.1	28.2 ± 1.8	23.2 ± 2.2	
	182	26.2 ± 3.6	26.0 ± 3.5	24.4 ± 3.1	20.8^*^± 1.9	
Creat mg/dL	−70	1.26 ± 0.28	1.28 ± 0.19	1.32 ± 0.38	1.22 ± 0.18	0.9–2.2
	−7	1.24 ± 0.26	1.42 ± 0.08	1.20 ± 0.12	1.24 ± 0.13	
	100	1.22 ± 0.08	1.52^*^± 0.08	1.56^*^± 0.21	1.28^*^± 0.13	
	182	1.18 ± 0.22	1.34 ± 0.11	1.12 ± 0.13	1.04 ± 0.17	
Total bilirubin mg/dL	−70	0.02 ± 0.04	0.00 ± 0.00	0.00 ± 0.00	0.02 ± 0.04	0–0.1
	−7	0.00 ± 0.00 n	0.00 ± 0.00 n	0.00 ± 0.00 n	0.00 ± 0.00 n	
	100	0.00 ± 0.00 n	0.00 ± 0.00 n	0.00 ± 0.00 n	0.00 ± 0.00 n	
	182	0.00 ± 0.00 n	0.00 ± 0.00 n	0.00 ± 0.00 n	0.00 ± 0.00 n	
Bile acids μg/mL	−7	0.069 ± 0.093	0.138 ± 0.137	0.159 ± 0.259	0.081 ± 0.028	0–2.04$
	100	1.321 ± 2.551	0.132 ± 0.247	0.189 ± 0.355	0.138 ± 0.142	
	182	0.173 ± 0.317	0.154 ± 0.135	0.216 ± 0.127	0.269 ± 0.134	
AST U/L	−70	23.8 ± 2.8	27.8 ± 5.0	28.0 ± 7.3	26.2 ± 5.6	7–38
	−7	28.4 ± 4.8	30.4 ± 8.9	24.0 ± 506	24.4 ± 3.7	
	100	25.0 ± 7.0	24.8 ± 1.9	31.0 ± 9.4	24.4 ± 2.9	
	182	28.2 ± 7.1	25.6 ± 3.8	22.4 ± 6.0	22.4 ± 2.5	
ALT U/L	−70^#^	73.2 ± 10.8	102.8 ± 47.3	82.2 ± 25.0	95.8 ± 25.6	25–97
	−7	65.2 ± 9.6	71.6 ± 18.9	45.4^*^± 6.9	57.6 ± 6.7	
	100	59.8 ± 15.0	57.8 ± 7.8	50.6 ± 5.8	47. ± 8.1	
	182	65.4 ± 17.8	52.4^*^± 10.4	42.6^*^± 6.5	48.6^*^± 7.6	
ALP U/L	−70	24.4 ± 11.2	20.8 ± 4.1	24.8 ± 8.6	20.2 ± 12.1	0–45
	−7	20.4 ± 4.7	16.6 ± 3.3	24.0 ± 9.4	19.0 ± 5.8	
	100	20.6 ± 4.8	16.6 ± 5.6	19.0 ± 5.4	15.2 ± 4.1	
	182	14.0 ± 5.5	17.8 ± 2.5	18.4 ± 3.8	17.4 ± 5.4	
SDH U/L	−7	4.74 ± 3.06	5.30 ± 3.91	1.44 ± 0.88	5.26 ± 4.59	3.9–7.7$
	100	1.18 ± 2.42	0.08 ± 0.18	0.00 ± 0.00	0.70 ± 0.64	
	182	5.60 ± 2.70	3.90 ± 1.12	4.32 ± 2.76	3.78 ± 0.79	
ALB g/dL	−70	3.56 ± 0.05	3.58 ± 0.19	3.42 ± 0.16	3.62 ± 0.30	2.8–3.9
	−7	3.18 ± 0.26	3.58^**^± 0.04	2.90 ± 0.14	3.44^**^± 0.15	
	100	3.26 ± 0.11	3.44 ± 0.26	3.40 ± 0.23	3.42 ± 0.11	
	182	3.18 ± 0.11	3.20 ± 0.12	3.06 ± 0.23	3.28 ± 0.11	
A/G	−70	1.10 ± 0.07	1.14 ± 0.09	1.06 ± 0.05	1.14 ± 0.13	0.45–1.45$
	−7	0.88 ± 0.08	0.92 ± 0.04	1.00 ± 0.10	0.94 ± 0.15	
	100	0.90 ± 0.00	0.96^*^± 0.05	1.06^*^± 0.05	0.98^*^± 0.08	
	182	0.96 ± 0.09	1.02 ± 0.08	1.12 ± 0.15	1.10 ± 0.07	
Total protein g/dL	−70	6.80 ± 0.32	6.72 ± 0.18	6.60 ± 0.38	6.84 ± 0.31	6.0–7.9
	−7	6.76 ± 0.38	7.42^*^± 0.26	5.88^*^± 0.66	7.10 ± 0.29	
	100	6.90 ± 0.31	7.02 ± 0.37	6.66 ± 0.58	6.90 ± 0.35	
	182	6.44 ± 0.29	6.34 ± 0.25	5.88 ± 0.79	6.24 ± 0.22	
Na^+^ mEq/L	−70	153.4 ± 1.5	153.6 ± 1.5	154.0 ± 1.9	154.0 ± 2.2	146–156
	−7	145.6 ± 7.0	154.6^**^± 1.3	137.0 ± 3.8	154.4^**^± 2.8	
	100	152.2 ± 2.3	153.4 ± 1.1	152.8 ± 1.6	152.8 ± 3.6	
	182	147.0 ± 1.4	148.4 ± 1.1	140.6 ± 3.8	142.8^*^± 2.8	
K^+^ mEq/L	−70	4.38 ± 0.26	4.28 ± 0.36	4.32 ± 0.30	4.20 ± 0.12	3.7–6.1
	−7	5.16 ± 0.90	5.14 ± 0.38	4.42 ± 0.31	5.16 ± 0.47	
	100	5.18 ± 0.36	5.38 ± 0.48	5.46 ± 0.42	5.06 ± 0.47	
	182	5.12 ± 0.41	5.54 ± 0.36	4.78 ± 0.16	5.14 ± 0.31	
Calc mg/dL	−70	9.38 ± 0.23	9.56 ± 0.47	9.38 ± 0.30	9.56 ± 0.24	8.7–11.7
	−7	9.20 ± 0.79	9.94^*^± 0.36	8.22 ± 0.38	9.98^*^± 0.29	
	100	9.54 ± 0.22	9.78 ± 0.51	9.40 ± 0.45	9.54 ± 0.24	
	182	9.18 ± 0.25	9.60 ± 0.22	8.62^*^± 0.42	9.08 ± 0.13	
Phos mg/dL	−7	4.60 ± 0.31	4.96 ± 0.48	3.60^**^± 0.20	4.90 ± 0.66	3.0–6.1
	100	4.94 ± 0.48	4.08^*^± 0.29	4.18^*^± 0.54	4.18^*^± 0.58	
	182	5.10 ± 0.80	4.48^*^± 0.63	4.06^*^± 0.45	4.34^*^± 0.15	
Cl^−^ mEq/L	−70	118.4 ± 2.6	118.2 ± 1.6	119.2 ± 0.8	118.8 ± 1.8	115–130
	−7	115.2 ± 5.8	122.8 ± 0.4	109.6 ± 3.8	122.4 ± 1.1	
	100	122.4 ± 1.5	123.0 ± 0.7	122.2 ± 1.1	123.0 ± 2.3	
	182	117.4 ± 1.5	119.6 ± 1.1	113.2 ± 3.0	114.4^*^± 1.7	
Blood taurine nmol/mL	−7/−6	451.6 ± 82.5	395.8 ± 103.1	379.8 ± 52.6	441.2 ± 67.6	275–701$$
	100/101	332.4 ± 118.3	365.4 ± 120.7	302.2 ± 130.5	317.4 ± 79.8	
	182	438.6 ± 97.2	424.4 ± 85.1	338.2 ± 66.3	435.4 ± 57.0	

*AFD1, anti-Fel d 1 IgY; Creat, Creatinine; AST, Aspartate transferase; ALT, Alanine transferase; ALP, Alkaline phosphatase; SDH, Sorbitol Dehydrogenase; ALB, Albumin; A/G, Albumin/Globulin Ratio; Na^+^, Sodium; K^+^, Potassium; Calc, Calcium; Cl^−^, Chloride; Phos, Phosphorus*.

* *Significant at P < 0.05 when compared to the control group within a study day*.

** *Significant at P < 0.01 when compared to the control group within a study day*.

# *Day−70 data obtained at Vendor location prior to shipment to study site*.

*SD, Standard deviation; n, Data not appropriate for statistical analysis*;

$ *Kaneko (24)*;

$$ *UC Davis (21)*.

On Day 100, the statistically lower mean bile acid concentrations seen in the 7 and 66 ppm male groups had no relation to dietary AFD1, because low bile acids are expected in clinically healthy, fasted mammals [reference range: 0–2.04 μg/mL; (24)] and an increased fasting serum bile acid level is indicative of a decreased hepatic function (26). Conversely, it should be noted that control female Cat 20 had a bile acid concentration >5.72 μg/mL on Day 100^2^. The higher value in this female may have been the result of a longer fasting period and was not accompanied by concurrent changes in other hepatobiliary parameters such as GGT (reported at 0 for all controls and AFD1-feed groups and time points; data not shown) or ALP (Table 8) and was therefore attributed to normal biologic variation (26).

On Day 100, the mean ALT activity was statistically significantly higher in the 66 ppm male group relative to the respective control male group because of mildly higher activity (>100 U/L) in Cat 23; the ALT value for this male was also mildly higher on Day 182, but the mean value for the 66 ppm male group was not statistically significantly different from controls. The ALT values for the groups were significantly greater than the control group prior to the start of (Day−7), which may have influenced statistical significance at Day 100 and indicates a lack of biological significance. No statistically significant changes were seen when comparing the means of other hepatocellular parameters (AST, GGT, SDH, bile acids, and total bilirubin) from male AFD1-fed groups to those of respective controls. The statistically significant difference in mean ALT for the 66 ppm male group at Day 100 (Table 7) was considered to be within normal biologic variation and not related to the AFD1-containing ingredient in the feed. On Day 182, the mean ALT values were significantly lower in all the AFD1-fed female groups, which is not considered a toxicological effect, were not of a AFD1-responsive nature and remained within normal reference values [25–97 U/L; (14)] except for the 7 ppm group on Day−70 (102.8 ± 47.3 U/L), which was not related to administration of the AFD1 diet.

SDH activity generally ranges from ~0 to 15 U/L in clinically healthy cats (27). On Day 182, the 39 ppm male group had a statistically higher mean SDH activity than concurrent control. The minimally higher mean activity was caused by individual values (>10 U/L) in male cats 2 (11.8 U/L) and 5 (10.7 U/L). This minimally higher mean value in the 39 ppm male group was not attributed to the AFD1 ingredient because similar individual values (>10 U/L) were observed in three females on acclimation Day−7 [7 ppm group Cat 39 (11.5 U/L) and 66 ppm group Cats 26 (10.2 U/L) and 29 (10.1 U/L)], the values were within or below reference values and they were therefore considered to be within normal biologic variation. Increases in SDH above normal ranges is a potential concern for hepatocyte injury, but lower than typical SDH levels is not considered an adverse event.

On Day 100, statistically higher mean creatinine concentrations were seen in all male and female AFD1-fed groups. The increases in these groups were not observed on Day 182 (with continued feeding), were within reference ranges for healthy animals [0.9–2.2 mg/dL; (14)] and were not AFD1 level dependent (as Creat concentrations did not increase with increasing levels of AFD1 consumption); changes in Creat levels were considered incidental to AFD1 consumption.

Statistically significant differences in mean A/G ratios have no biologic meaning without noteworthy changes in albumin or calculated total globulin concentrations. The significantly higher mean A/G ratios on Day 100 for the 39 ppm male group and all female groups fed AFD1 simply reflected the minimally higher proportion of albumin relative to globulin. There were no biologically relevant increases in mean albumin or decreases in mean globulin concentrations in the same groups at those intervals. Additionally, the values were considered to be within normal biologic variability [reference range of ALB at 2.8–3.9 g/dL and globulin at 2.6–5.1 g/dL; (14)], comparable to values obtained prior to test ingredient feeding. There is substantial biological variation within the cat population that is apparent in hematologic and biochemistry values (17), which may explain the variation in these values seen in this study. However, the incorporation of the test substance had no clear AFD1 level-responsive effect that was toxicologically relevant. Similar to the male cats, no effect of dietary AFD1 was found on whole blood taurine levels in the female cats (Table 8) in any AFD1-fed group, when compared to the control group (*P* > 0.05).

On Day 182, a significantly lower mean calcium concentration was observed in the 39 ppm female group (8.62 ± 0.42 mg/dL); the difference from the respective control group was considered incidental to the AFD1 ingredient because of the lack of a AFD1-related response and the calcium levels nearly within published reference ranges [8.7–11.7 mg/dL; (14)]. Hypocalemia may be an indicator of renal disease (14), but in this study there were no significant changes in serum total protein, serum urea nitrogen, or serum creatinine concentrations in the same study group at the same time point, or in the 66 ppm female group, confirming a lack of test AFD1-response effect and changes in other parameters indicative of renal issues that would be expected if the effect were to be test article-related. Although statistically significant, the minimally lower mean inorganic phosphorus values seen in all AFD1-fed female groups on Days 100 and 182 were of no biologic relevance (approximately one half of the mean calcium concentrations), not AFD1 level dependent, within published reference ranges [3.0–6.1 mg/dL; (14)] and considered incidental. Clinically significant hypophosphatemia is uncommon in cats and may be a health issue if prolonged levels of <2.5 mg/dL occur (28), which did not occur in this study.

The mean sodium concentration was statistically significantly lower in the 66 ppm female group on Day 182 (Table 8). This minimal decrease was considered incidental to the AFD1 ingredient because of the lack of a clear AFD1-related response; in fact, the mean value for the 39 ppm female group was lower, but not significantly different, from control females [reference range: 146–156 mEq/L sodium; (14)] at both Day−7 and at Day 182, indicating variable serum sodium concentrations for this study. Hyponatremia (considered ~11–15 units below reference ranges) did not occur in this study (29).

The statistically significant decrease in mean chloride noted for the 66 ppm female group on Day 182 was considered incidental to the AFD1 ingredient because of the lack of a clear AFD1 level-related response effect. Similar to sodium, the mean chloride value was lower in 39 ppm females than in 66 ppm females, but the difference was not statistically significantly different from the mean control female value. Conversely, there were statistically significant increases in chloride levels on Day−7 in the 7 and 66 ppm male groups; however, these concentrations were within reference ranges [115–130 mEq/L; (14)]. Phosphorus concentrations in the male treatment groups were significantly greater than the control group at Day−7 (prior to study start), but were within reference ranges [3.0-6.1 mg/dL; (14)] and no significant effects were noted in the males for this parameter for the rest of the study. Phosphorus concentrations were significantly decreased (*P* < 0.05) in the 7, 39, and 66 ppm female groups on Days 100 and 182 of the study, but again these concentrations were within publicly available reference ranges [3.0–6.1 mg/dL; (14)] and the slight decreases were not considered toxicologically relevant and did not parallel consumption of increasing amounts of AFD1; therefore, the changes in phosphorus concentrations were not considered AFD1-related. The changes in the clinical chemistry parameters were not indicative of significant toxicological action of AFD1 on the kidney, liver, or any other organs, as the significant changes in one parameter (such as the decreased ALT for the female AFD1 groups) is not correlated to a toxicological effect on the organ (i.e., liver) and was not consistent with other related parameters.

There was no effect of dietary AFD1 on any urinalysis parameters or urine microscopic sediment examination observations (data not shown). The urine was well-concentrated, and the results were generally unremarkable and similar among the control and test groups at each collection interval (Days−6, 100, and 182 of the study; data not shown).

## Conclusions

No biologically meaningful alterations were observed in the clinical chemistry, hematology, coagulation, or urinalysis parameters that could be attributed to dietary AFD1 provided to adult cats for 182 days (i.e., 26 weeks) at the levels tested. As with all analyses of clinical and hematological endpoints, there is a certain amount of intra-individual and inter-individual variation, such that a systematic assessment of the data is necessary to determine the health of the subject (30). Within this study there were variations in the individual cat clinical chemistry and hematology parameters, but there was no consistent effect that was outside published reference ranges indicating a toxicological response on the cats, when the parameters were evaluated as a whole (17, 30, 31). Similar to the results found in this feeding study, Satyaraj et al. (9) found variations in body weight occurring during consumption of the AFD1-containing diet (e.g., a general increase in body weight), but a reduction in the amount of food provided to the cats helped return the cats to baseline body weight. The increase in body weight indicates that the AFD1-containing diet was palatable in both studies and did not inhibit body maintenance. In addition, the decrease in active Fel d 1 levels in the cat hair reported in the Satyaraj et al. (9) study did not result in adverse effects that would require removal of any of the cats from the study, as only one of the 105 cats that consumed the AFD1-containing diet did not complete the study (the single cat was removed not for adverse effects from consuming the diet, but for having a “fractious personality”). An additional study by Satyaraj et al. (32) analyzed the effect of consumption of the AFD1 antibody by cats in reduction of salivary Fel d 1 antigen, finding the AFD1 significantly reduced active Fel d 1 saliva concentrations within 3 weeks of consumption in both a 6-week feeding trial and a 4-week feeding trial. The test diets contained a dried egg product calculated to provide ~8 ppm (on a dry matter basis) AFD1. The second trial utilized a treatment group and a control group, with 86% of the cats fed AFD1-containing diet showing a reduction in salivary active Fel d 1 of at least 20%, compared to only 38% of the control cats (32). The genotoxicity studies on the AFD1 ingredient show a lack of the potential to form mutagenic effects or chromosomal aberrations, as under the Delaney Clause of the Federal Food, Drug, and Cosmetic Act (FFD&C Act) food additives that have been found to induce cancer in humans or animals cannot be safety added to food.

Chicken eggs and egg products have been safely consumed for centuries. The use of concentrated IgY from chicken eggs is more recent but has also been shown to be safe in other species (33–37). This work evaluated the safety of Fel d 1-specific IgY antibody when fed to cats and evaluated the potential of this specific IgY to promote mutagenic or genotoxic effects. When added to dry cat food and provided to cats for 26 weeks, no adverse effects were observed that were attributed to AFD1 at the levels tested. The lack of mutagenic potential and the absence of chromosomal aberration formation in an OECD-compliant study confirms the lack of genotoxicity of the AFD1 ingredient. No biologically meaningful alterations were observed in the clinical pathology data that could be attributed to AFD1 in cat kibble for 26 weeks at the levels tested.

Sensitivities to cat-derived allergens is one of the leading reasons for relinquishment to shelters and serve as a barrier to cat adoption and ownership (38–43). Approximately 12.1% of the U.S. population over the age of 6 are estimated to be sensitized to cat allergens (44), and up to 26% of adult Europeans (45). Consistent avoidance of cats as household pets is not always practical, and allergen-specific subcutaneous immunotherapy for cat allergens does not always result in complete inhibition of conjunctival, nasal and bronchial allergy symptoms (46). A reduction in the amount of allergenic Fel d 1 released into the environment via shed hair and dander could reduce the allergen load in the environment while not changing a cat's natural Fel d 1 production or adversely affecting cat health. Reducing the allergen load has been shown to be beneficial for allergy sufferers (47, 48). The studies reported here show that the AFD1 ingredient is safe when incorporated into complete and balanced cat food.

## Data Availability Statement

Safety-related datasets generated for this study are included in the article/supplementary material.

## Ethics Statement

The animal study was reviewed and approved by Institutional Animal Care and Use Committee (IACUC). Written informed consent was obtained from the owners for the participation of their animals in this study.

## Author Contributions

DC and LT helped prepare the protocol, partially monitored the studies, and contributed in the preparation of the manuscript. RM helped to initiate and monitor the studies conducted, and prepared and edited the manuscript.

### Conflict of Interest

DC and LT are employees of Nestlé Purina PetCare Global Resources, Inc. RM was compensated for study monitoring and manuscript preparation.

## Footnotes

^1^Specific diet cats were previously consuming was not provided; however, spare cats were placed on Purina Savor Chicken and Rice diet if not enrolled in the study.

^2^Study measurements were obtained in SI units; a conversion factor of 2.44 was utilized to provide concentration in conventional units.
